# Interplay of actin nematodynamics and anisotropic tension controls endothelial mechanics

**DOI:** 10.1038/s41567-025-02847-3

**Published:** 2025-04-18

**Authors:** Claire A. Dessalles, Nicolas Cuny, Arthur Boutillon, Paul F. Salipante, Avin Babataheri, Abdul I. Barakat, Guillaume Salbreux

**Affiliations:** 1 Laboratoire d’Hydrodynamique (LadHyX), CNRS–École Polytechnique, Institut Polytechnique de Paris, Palaiseau, France; 2 Department of Biochemistry, University of Geneva, Geneva, Switzerland; 3 Department of Genetics and Evolution, University of Geneva, Geneva, Switzerland; 4 Laboratory for Optics and Biosciences, CNRS UMR7645, INSERM U1182, Institut Polytechnique de Paris, Palaiseau, France; 5 Cluster of Excellence Physics of Life, Technische Universität Dresden, Dresden, Germany; 6 Polymers and Complex Fluids Group, National Institute of Standards and Technology, Gaithersburg, MD USA

**Keywords:** Soft materials, Biological physics

## Abstract

Blood vessels expand and contract actively as they continuously experience dynamic external stresses from blood flow. The mechanical response of the vessel wall is that of a composite material: its mechanical properties depend on its cellular components, which change dynamically as the cells respond to external stress. Mapping the relationship between these underlying cellular processes and emergent tissue mechanics is an ongoing challenge, particularly in endothelial cells. Here we assess the mechanics and cellular dynamics of an endothelial tube using a microstretcher that mimics the native environment of blood vessels. The characterization of the instantaneous monolayer elasticity reveals a strain-stiffening, actin-dependent and substrate-responsive behaviour. After a physiological pressure increase, the tissue displays a fluid-like expansion, with the reorientation of cell shape and actin fibres. We introduce a mechanical model that considers the actin fibres as a network in the nematic phase and couples their dynamics with active and elastic fibre tension. The model accurately describes the response to the pressure of endothelial tubes.

## Abstract (web summary)

Blood flow through a vessel deforms vessel walls. Cells lining these walls sense the changes in pressure as blood flows and reorient their actin fibres in the direction of largest tension.

## Main

The hierarchical structure of the cardiovascular system matures after the onset of blood flow, starting from an initial microvascular meshwork in the embryo^1–3^. In an adult, small vessels regulate the blood flow actively through changes in their diameter to optimize tissue oxygenation. The deformation of these microvessels depends on the mechanical properties of their walls^4^. The endothelium, the main constituent of the thin wall of microvessels, is a composite material: a tubular assembly of connected cells, where each cell itself is an assembly of various biological components. Consequently, the overall mechanics of this living material emerges from both properties and interactions of its constituents—but neither are constant. Subcellular processes respond to external stresses, such as changes in wall tension, and alter the individual cells, thereby inducing dynamic adaptation in tissues in both physiological and pathological cases^5^.

Although these changes initiate at the smallest scales, they propagate to the largest structures. In this adaptation process as well as in tissue mechanics, the primary actors are the cytoskeleton and adhesion complexes, physically connecting cells to the substrate and to neighbouring cells^6–8^. Tissues exhibit viscoelastic behaviour, stemming from cytoskeletal elements, such as actin and intermediate filaments^9–12^, and from intercellular junctions^8,13,14^ and adhesions to the substrate^15,16^. Besides passively resisting deformations, the actomyosin network creates contractile active stresses. Force transmission at adhesions propagates these subcellular stresses to the tissue level. On long timescales, cellular rearrangements such as intercalation, division and apoptosis influence the rheology of tissues^7,17,18^.

Deciphering how subcellular processes and their regulation by force-sensing mechanisms are coupled across scales to give rise to active tissue mechanics is a current challenge. To that end, in vitro systems have become instrumental, with two main families: stretchers and mechanical testing platforms. Stretchers subject the substrate to controlled changes in length over time. Concomitant monitoring of the cellular response has led to the discovery of a host of mechanoadaptation mechanisms, such as the remodelling of actin fibres and junctions or cell stiffening^5,19–22^. In comparison, mechanical testing platforms, including systems such as micropipette aspiration or indentation, provide quantitative measurements of the material properties of cells and tissues^7,18,23,24^. Despite the remarkable advancements achieved with these systems, addressing the coupling between tissue mechanics and the multiscale dynamics of the mechanoresponsive components remains elusive, due to the difficulty of observing living tissue over the required spatial and temporal scales.

## Results

### Endothelial tubes exhibit actin-dependent elasticity

#### Anisotropic tension induced by luminal pressure

**Fig. 1:**
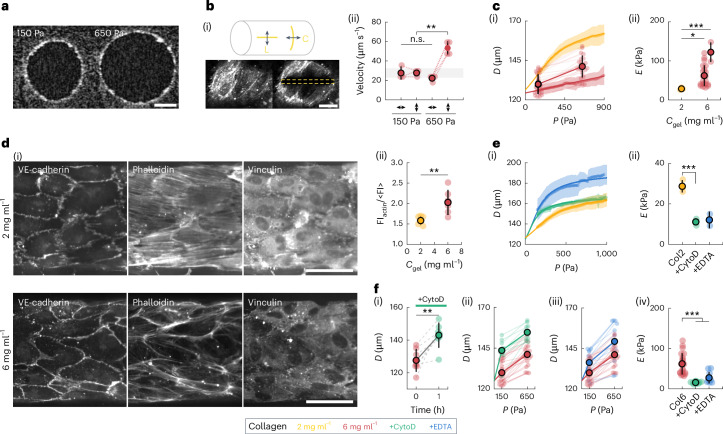
Endothelial tubes exhibit actin-dependent elasticity under luminal pressure. **a**, Optical coherence tomography images of the vessel cross-section showing an increase in radius during pressure increase. Scale bar, 50 μm. **b**, Schematic of laser ablation showing the two directions of ablation: longitudinal (L) and circumferential (C) (i). Fluorescence images of LifeAct-endothelial cells showing the endothelial actin network pre- and post-longitudinal ablation (the area of ablation is denoted in yellow), showing a rapid opening of the wound, which is characteristic of high tissue tension in the circumferential direction. Scale bar, 20 μm. **b**, Initial recoil velocity post-ablation for monolayers cultured on a 6 mg ml^−1^ collagen gel, showing an increase between the control (150 Pa) and stretched (650 Pa) channels, but only in the circumferential direction (ii). Ablations were performed in the minutes following the pressure increase for the stretched condition (*n* = 3). **c**, Channel diameter as a function of the luminal pressure (points) for monolayers cultured on a 2 mg ml^−1^ (yellow, *n* = 3) and 6 mg ml^−1^ (red) collagen gel, obtained either continuously with live imaging (chain of dots, *n* = 3) or at the beginning and end of pressure application (paired dots, *n* = 18), with the fitted analytical curves obtained from the strain-stiffening model (solid lines) (i). **c**, Inferred Young’s moduli of the endothelial tissue for the two collagen concentrations. For the 6 mg ml^−1^ concentration (red), data from the continuous measurement (right, *n* = 3) and the discrete two-point measurement (left, *n* = 18), matching the curves in **b**(ii), are separated for clarity (ii). **d**, Endothelium stained for VE-cadherin, phalloidin and vinculin for two collagen concentrations (i): 2 mg ml^−1^ (top) and 6 mg ml^−1^ (bottom). Fluorescence intensity of the actin stress fibres (normalized by the mean cell intensity) as a function of collagen concentration (*n* = 5 (2 mg ml^–1^) and *n* = 6 (6 mg ml^–1^)) (ii). **e**, Channel diameter as a function of luminal pressure for control monolayers (yellow, *n* = 3) and monolayers treated with cytochalasin D (green, *n* = 3) and EDTA (blue, *n* = 2), cultured on a 2 mg ml^−1^ collagen gel (i). Inferred Young’s moduli of control (*n* = 3) and endothelia treated with cytochalasin D (*n* = 3) and EDTA (*n* = 2), cultured on a 2 mg ml^−1^ collagen gel (ii). **f**, Channel diameter as a function of time just after treatment with cytochalasin D (at *t* = 0), for monolayers cultured on a 6 mg ml^−1^ collagen gel (*n* = 7) (i). Channel diameter as a function of luminal pressure for control monolayers (red, *n* = 18) and monolayers treated with cytochalasin D (green, *n* = 9) and EDTA (blue, *n* = 12), cultured on a 6 mg ml^−1^ collagen gel ((ii) and (iii)). Inferred Young’s moduli of control (*n* = 18) and endothelia treated with cytochalasin D (*n* = 9) and EDTA (*n* = 12), cultured on a 6 mg ml^−1^ collagen gel (iv). Source data

**Extended Data Fig. 1:**
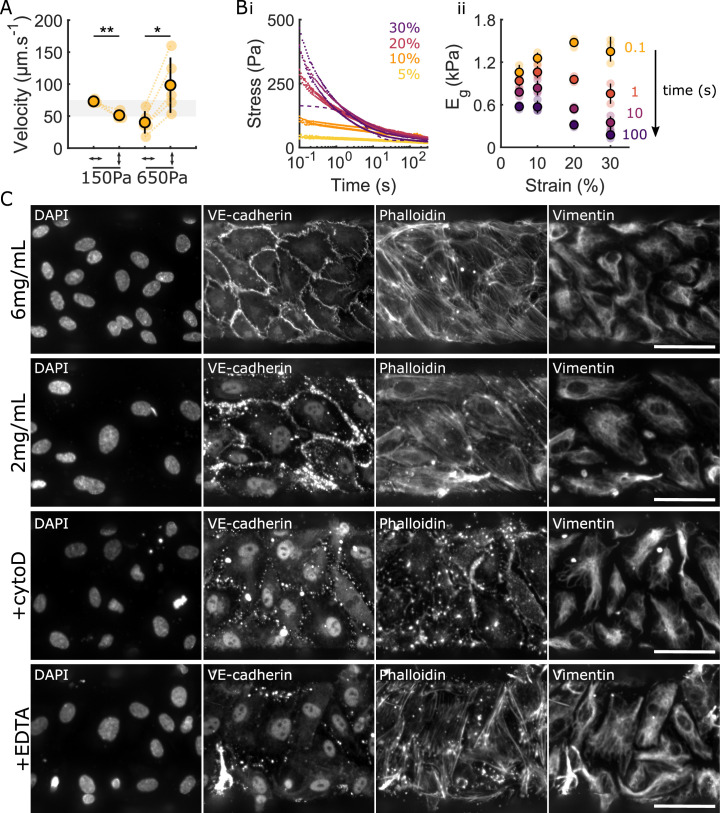
Endothelial tubes exhibit actin-dependent elasticity under luminal pressure. **A** Initial recoil velocity post ablation for monolayers cultured on a 2 mg.mL^−1^ collagen gel, showing an increase between the control (150Pa, n=3) and stretched (650Pa, n=5) channels, but only in the circumferential direction. **Bi** Shear stress in collagen gels, measured in a rheometer, as a function of time (dots), after a strain step of varying amplitude, ranging from 5 (yellow) to 30% (purple). n=3 in each strain condition. Stress curves show a decay, indicating stress relaxation, enhanced for higher strains. The stress relaxation curves are fitted with an exponential, stemming from a Maxwell viscoelastic model with a residual stress. One example is shown for a strain of 30 % (dashed line). **Bii** Young’s moduli extracted from the stress relaxation curves as a function of the applied strain at different timepoints after the strain step, from 0.1 s (yellow) to 100 s (purple), showing a switch from strain-stiffening to strain-softening as a function of time. **C** Endothelium stained for DAPI, VE-cadherin, phalloidin and vimentin one minute after the application of the 1000 Pa pressure, for the two collagen concentrations: 2 mg.mL^−1^ (n=3) and 6 mg.mL^−1^ (n=3), and with the cytochalasinD (n=3) or EDTA (n=2) treatment, on the soft gel. Scale bar 50 *μ*m. Source data

Here we use our previously developed microstretcher, mimicking the native environment of blood vessels^25^, to impose tension on a tubular endothelium templated within a soft collagen hydrogel through a physiological increase in luminal pressure (Fig. 1a and Supplementary Video 1). To assess tissue tension, we performed laser ablation in monolayers expressing the actin reporter LifeAct, along the longitudinal and circumferential directions (Fig. 1b(i) and Supplementary Video 2), at the low pressure used for monolayer culture (150 Pa) and in the minutes following the pressure increase (650 Pa). The recoil velocity post-ablation is thought to increase with tissue tension and to decrease with tissue viscosity or elasticity^26,27^. The recoil velocity is doubled when the pressure is increased to 650 Pa, but only in the circumferential direction (Fig. 1b(ii) and Extended Data Fig. 1a), indicating an increase in circumferential tension. In addition, in monolayers on low-concentration hydrogels, the recoil velocities are higher (Extended Data Fig. 1a). As they are subjected to the same imposed tension, the viscoelastic properties of endothelia differ between soft and stiffer gels. Together, these results demonstrate a switch from an isotropic to an anisotropic tension on pressure increase and a substrate-dependent monolayer viscoelasticity.

#### Collagen gel mechanics

We then investigated the mechanical properties of the collagen gel to evaluate its mechanical contribution to the tube dynamics. The response to strain steps ranging from 5% to 30% of bulk collagen gels at a concentration of 6 mg ml^–1^ was measured using a plate rheometer (Extended Data Fig. 1b(i)). The stress in the gel relaxes in time (Extended Data Fig. 1b(i))^28,29^. For smaller strains of 5–10%, Young’s modulus relaxes to around 1 kPa after a minute of strain application. As the strain increases, the gel exhibits initial strain stiffening, which turns into strain softening after ~10 s, due to strain-enhanced stress relaxation^30^. As a result, Young’s moduli values drop below 1 kPa for strains of 20–30% after 100 s of strain application (Extended Data Fig. 1b(ii)). Consequently, the contribution of gel-resisting pressure is small compared with the applied pressure in the lumen (Supplementary Section 2.1.2). We, therefore, neglect the mechanical contribution of the hydrogel hereafter.

#### Substrate-dependent stiffness of endothelial tubes

To quantitatively measure tissue stiffness, we recorded the strain–stress curves in a physiological range of stress. We applied an external tension on the tissue by increasing the pressure continuously from 150 Pa to 1,000 Pa in one minute, and measured the deformation of the channel, with live imaging (Fig. 1c,e and Supplementary Video 3) or at the beginning and end of pressure application (Fig. 1f). The maximum pressure was chosen to approximate the pressure in native capillaries, where the vessel wall is composed of a single cell layer, of around 1 kPa (ref. ^31^).

We find the tissue stiffness to be around 0.13 N m^–1^ on the softer gel and 0.26 to 0.4 N m^–1^ on the stiffer gel, corresponding to Young’s moduli of 30 kPa and 50–120 kPa (Fig. 1c(ii)), confirming the substrate-dependent tissue stiffening observed with laser ablations and consistent with previous findings^32^. These Young’s moduli values are similar to those reported for suspended epithelial tissues^33^ and are an order of magnitude higher than the value of the collagen gels found above, confirming its negligible contribution. In addition, the deformation–pressure curves show a strain-stiffening behaviour, with a threshold strain of approximately 20% separating a linear regime at low pressures and a saturating regime at large pressures, which can be captured using a Gent model (Fig. 1c(i))^21,32^.

#### Subcellular determinants of endothelium elasticity

To understand the biological origin of the substrate-dependent mechanics, we imaged the actin network and its anchoring points. More prominent actin filaments and larger focal adhesions (FAs) are found on stiffer gels (Fig. 1d). Actin stress fibres appear sensitive to substrate density or mechanical stiffness, and their reinforcement could underlie the substrate-dependent stiffening^33,34^.

To further probe the mechanical contribution of actin and cell–cell junctions, we treated the monolayers with either cytochalasin D (an inhibitor of actin polymerization) or EDTA (a perturbator of adherens junctions (AJs)) (Extended Data Fig. 1c). Both treatments lead to a substantial increase in monolayer deformation for both collagen concentrations (Fig. 1e(i),f(i),(ii)), characteristic of tissue softening. Young’s modulus drops to 10–20 kPa on actin depolymerization (Fig. 1e(ii),f(iv)), consistent with previous reports^21,33^, for both gel concentrations, confirming that actin underlies the adaptation to substrate properties. In addition, the maximum strains of actin-depleted monolayers are identical to untreated monolayers for a low collagen concentration (Fig. 1e(i)), suggesting that another cytoskeletal element controls the large deformation regime. We speculate that it could be intermediate filaments, intact in cells with depolymerized actin (Extended Data Fig. 1c), as reported previously^10,35,36^. Perturbing AJs decreases the effective Young’s modulus of the endothelium to 15 kPa and 50 kPa on the 2 mg ml^−1^ and 6 mg ml^−1^ collagen concentrations, respectively (Fig. 1e(ii),f(iv)). The tissue probably has some mechanical contribution despite being morcellated (Extended Data Fig. 1c), as these effective moduli are much higher than that of a bare gel (50–100 Pa for the 2 mg ml^−1^ collagen and 600–1,000 Pa for the 6 mg ml^−1^ collagen)^37^. Individual cells may induce local stiffening due to their adhesions to the underlying matrix. The final strain increases (Fig. 1e(i),f(iii)), probably due to the stretching of bare collagen between cells.

### Cells and actin stress fibres align in the tension direction

We then sought to study endothelial tissue mechanics and long-term adaptation to a change in luminal pressure, a phenomenon occurring in the native vasculature, for instance, at the onset of blood flow in the embryo or due to pathologies in the adult^3,38,39^. We, therefore, subjected the endothelial tube, formed at 150 Pa, to a fixed luminal pressure of 650 Pa for several days and monitored the tissue and cellular responses. The order of magnitude of this pressure increase mimics the initial pressurization of the native vascular network in embryos^3^ and the 400–900 Pa increase in capillary pressure found in hypertensive patients^38,39^.

**Fig. 2:**
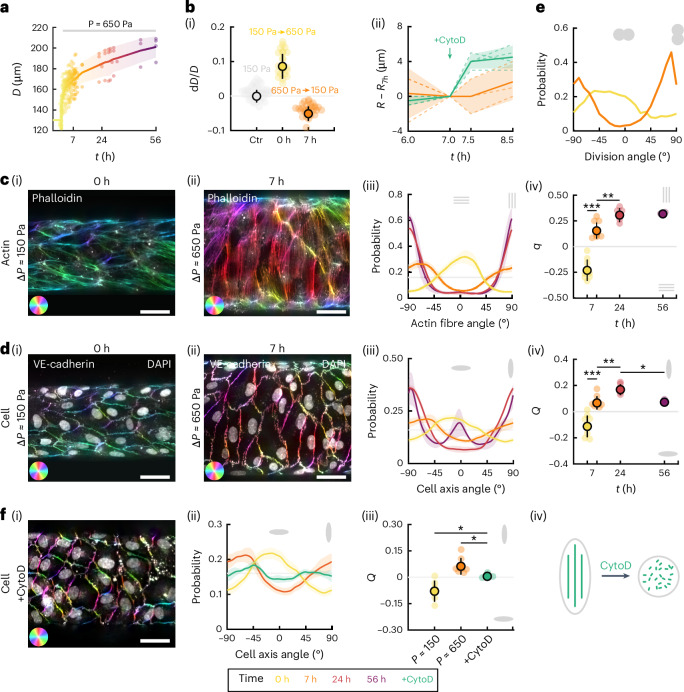
Cells dynamically align in the tension direction via an active actin-dependent process. **a**, Channel diameter as a function of time after the pressure increase (*t* = 0), colour coded for time (*n* = 6). **b**, Relative diameter change when increasing pressure from 150 Pa to 650 Pa (yellow, *n* = 21) and when decreasing pressure back to 150 Pa after 7 h later (orange, *n* = 21) (i). The diameter fluctuations at 150 Pa are shown in grey as a reference (Ctr). Evolution of the channel diameter between 6 h and 8.5 h for control monolayers (orange, *n* = 3) and for monolayers treated with cytochalasin D at *t* = 7 h (green, *n* = 3) under a pressure of 650 Pa, showing a sudden diameter increase due to actin depolymerization (ii). **c**, Endothelium stained for phalloidin at *t* = 0 h under 150 Pa (i) and after *t* = 7 h under 650 Pa (ii), where the orientation of the actin stress fibres is colour coded. Evolution of the probability distribution of the actin stress fibre orientation (iii) and the associated nematic order parameter *q* (iv) at 0 h (yellow, *n* = 8), 7 h (orange, *n* = 9), 24 h (red, *n* = 5) and 56 h (purple, *n* = 2). **d**, Endothelium stained for VE-cadherin at *t* = 0 h under 150 Pa (i) and after *t* = 7 h under 650 Pa (ii), with the orientation of the junctions colour coded. Nuclei are overlaid in white. Evolution of the probability distribution of the cell orientation (iii) and the associated nematic order parameter *Q* (iv) at 0 h (yellow, *n* = 8), 7 h (orange, *n* = 7), 24 h (red, *n* = 5) and 56 h (purple, *n* = 2). **e**, Probability distribution of the division orientation for monolayers, measured at *t* = 7 h, under low pressure (Δ*P* ≈ 150 Pa, yellow) and high pressure (Δ*P* ≈ 650 Pa, orange). **f**, Cytochalasin-D-treated monolayer stained for VE-cadherin after 7 h of pressure showing round cells (i). Evolution of the probability distribution of the cell orientation (ii) and the associated nematic order parameter *Q* (iii) before pressure increase (150 Pa, *n* = 8), and after 7 h of high pressure for the control (650 Pa, *n* = 7) and cytochalasin-D-treated (CytoD, *n* = 3) monolayers. Schematic showing round cells after actin depolymerization by cytochalasin D treatment, despite the circumferential stretching force (iv). Scale bar, 50 μm. Source data

First, the diameter increases instantaneously due to the rapid pressure increase. Over the next 56 h, despite the fixed pressure, the diameter increases continuously, showing a fluid-like creeping behaviour (Fig. 2a). Decreasing the pressure back to 150 Pa after 7 h of excess pressure application results in a diameter decrease, one minute after pressure release (Fig. 2b(i)), validating the presence of tension in the tissue. The diameter does not, however, recover its original value, consistent with the dissipation of stresses in the tissue and the surrounding viscoplastic hydrogel. After cytochalasin D application at 7 h, the diameter increases abruptly (Fig. 2b(ii)), probably due to softening of the endothelial tube induced by actin depolymerization (Fig. 1e,f). This further supports the notion that tension in the tissue is resisting the applied pressure during the entire duration of the experiment.

**Extended Data Fig. 2:**
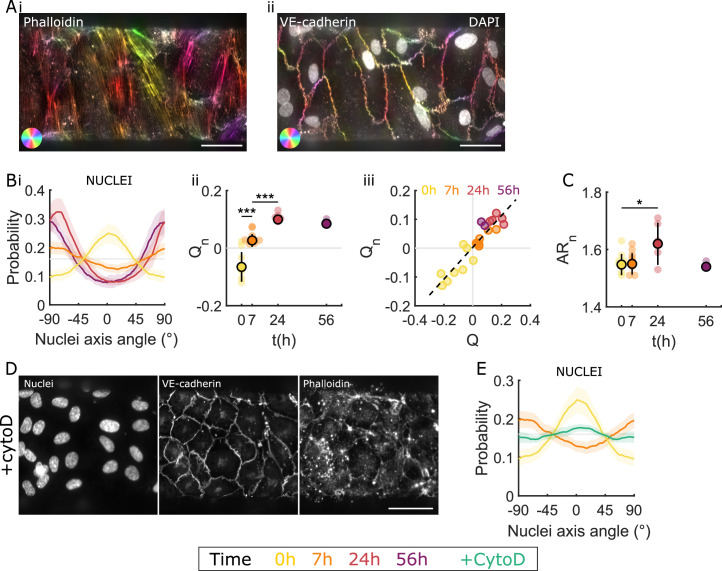
Cells dynamically align in the tension direction via an active actin-dependent process. **A** Endothelium stained for phalloidin (**i**) and VE-cadherin (**ii**) at *t* = 24 h under 650 Pa, with the orientation of the actin stress fibers and junctions color coded. Nuclei are overlaid in white (**ii**). **B** Evolution of the probability distribution of the nuclei orientation (**i**) and the associated nematic order parameter *Q*_n_ (**ii**) at 0 (yellow, n=8), 7 (orange, n=9), 24 (red, n=5) and 56 (purple, n=2) hours. The sign of *Q*_n_ denotes the direction of the orientation and the absolute value its strength. **Biii** *Q*_n_ as a function of the cell elongation order parameter *Q*, showing a linear empirical correlation, color coded for time (0h yellow, 7h orange, 24h red and 56h purple, same data as in Fig. 2div, Extended Data Fig. 2Bii). **C** Mean nucleus aspect ratio as a function of time. **D** Cytochalasin-treated monolayer stained for nuclei, VE-cadherin and phalloidin after 7h of pressure showing round cells, the presence of cell-cell junctions and the absence of actin fibers. Scale bar 50 *μ*m. **E** Evolution of the probability distribution of the nuclei orientation before the pressure increase (*ΔP* ≃ 150Pa, yellow, n=8), and after 7h of high pressure for the control (*ΔP*≈650Pa, orange, n=7) and cytochalasin-treated (green, n=3) monolayers. Source data

To link the dynamics of subcellular elements to the observed tissue flow, we imaged the actin network, cell–cell junctions and nuclei over time. During the assay, the actin cytoskeleton reorganizes from a longitudinal orientation to prominent stress fibres oriented in the circumferential direction (Fig. 2c(i)–(iii), Extended Data Fig. 2a(i) and Supplementary Videos 4, 6 and 8). Endothelial cells and nuclei elongations follow the same dynamic pattern (Fig. 2d(i)–(iii), Extended Data Fig. 2a(ii),b(i) and Supplementary Videos 5 and 7). In addition, the orientation of cell divisions switches from longitudinal at Δ*P* ≈ 150 Pa to circumferential at Δ*P* ≈ 650 Pa, aligning with the cell elongation axis (Fig. 2e).

We introduce the order parameters *q*, *Q* and *Q*_n_ characterizing the circumferential order and orientation of actin stress fibres, cell shapes and nuclei, respectively (Methods and Supplementary Information). Their sign indicates whether the elements are preferentially circumferential (>0) or longitudinal (<0), whereas their magnitude indicates the strength of this alignment. We find that *q*, *Q* and *Q*_n_ show significant differences between successive time points at 0 h, 7 h and 24 h, as the actin stress fibres, cells and nuclei progressively reorient (Fig. 2c(iv)d(iv) and Extended Data Fig. 2b(ii)). Nuclei elongation follows cell elongation (Extended Data Fig. 2b(iii),c).

### Cell elongation and alignment are actin-dependent processes

To disentangle whether cell elongation results from direct deformation by anisotropic stretch or from an active process, we treated the cells before the pressure increase with cytochalasin D to depolymerize actin. AJs are still present right after the treatment and after 7 h of pressure application (Fig. 2f(i) and Extended Data Fig. 2d). Cytochalasin-D-treated monolayers display round and randomly oriented cells (Fig. 2f(i)–(iv)) and randomly oriented nuclei (Extended Data Fig. 2e), showing that the cell elongation observed in this experiment is an active process, requiring an intact actin cytoskeleton.

### Actin alignment requires cell–cell junctions and FAs

**Fig. 3:**
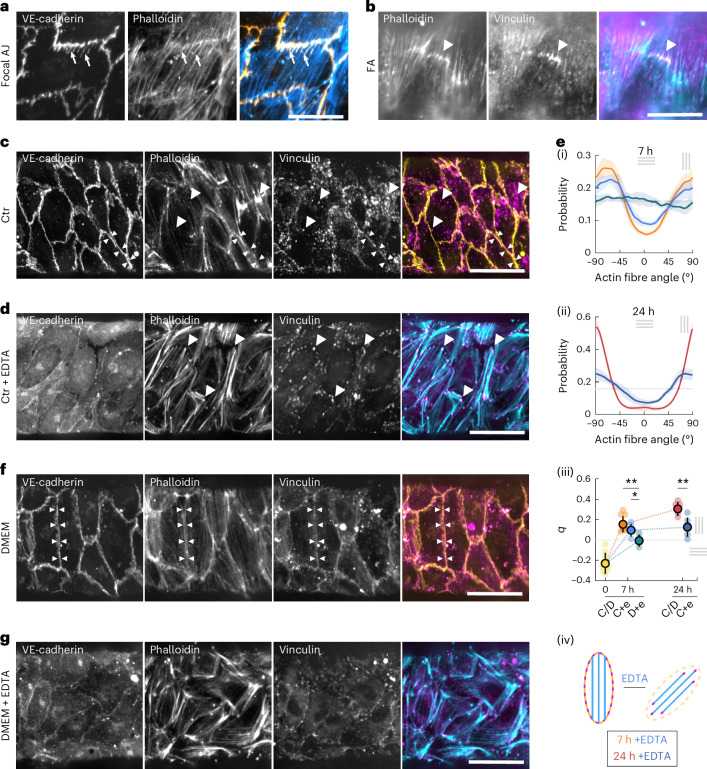
Cell–cell junctions and FAs are necessary for actin alignment. **a**, Endothelium stained for VE-cadherin (yellow) and phalloidin (cyan) after 7 h of stretch, showing a focal AJ with transendothelial actin fibre association (arrows). **b**, Endothelium stained for phalloidin (cyan) and vinculin (magenta) after 7 h of stretch at Δ*P* ≈ 650 Pa, showing a line of clustered FA with actin fibres anchoring (arrowhead). Scale bar, 20 μm (**a** and **b**). **c**, Control endothelium stained for VE-cadherin (yellow), phalloidin and vinculin (magenta) after 7 h of stretch, showing vinculin association to FAs at the end of actin stress fibres (arrowheads) and to AJs with parallel actin stress fibres (double arrowheads). **d**, EDTA-treated endothelia stained for VE-cadherin, phalloidin (cyan) and vinculin (magenta) after 7 h of stretch, showing vinculin association to FAs at the ends of actin stress fibres (arrowheads). **e**, Probability distribution of the actin stress fibres at 7 h (i) and 24 h (ii) for control (orange, *n* = 7 (7 h); red, *n* = 5 (24 h)) and EDTA-treated endothelia in the control medium (blue, *n* = 5 (7 h) and *n* = 6 (24 h)) or DMEM (teal, *n* = 3 (7 h)). Nematic order parameter *q* of the actin stress fibres for control (orange-red), in standard medium (label C) or DMEM medium (label D) and EDTA-treated endothelia (label +e), with the nematic order parameter *q* at 0 h (yellow, *n* = 8) (iii). Schematic of a cell before and after treatment with EDTA, with the actin anchoring switching from junctions to FAs (iv). **f**, DMEM-cultured endothelium stained for VE-cadherin (yellow), phalloidin and vinculin (magenta) after 7 h of stretch, showing vinculin association to AJs with parallel actin stress fibres (double arrowheads). **g**, DMEM-cultured and EDTA-treated endothelia stained for VE-cadherin, phalloidin (cyan) and vinculin (magenta) after 7 h of stretch. Scale bar, 50 μm (**c**, **d**, **f** and **g**). Source data

**Extended Data Fig. 3:**
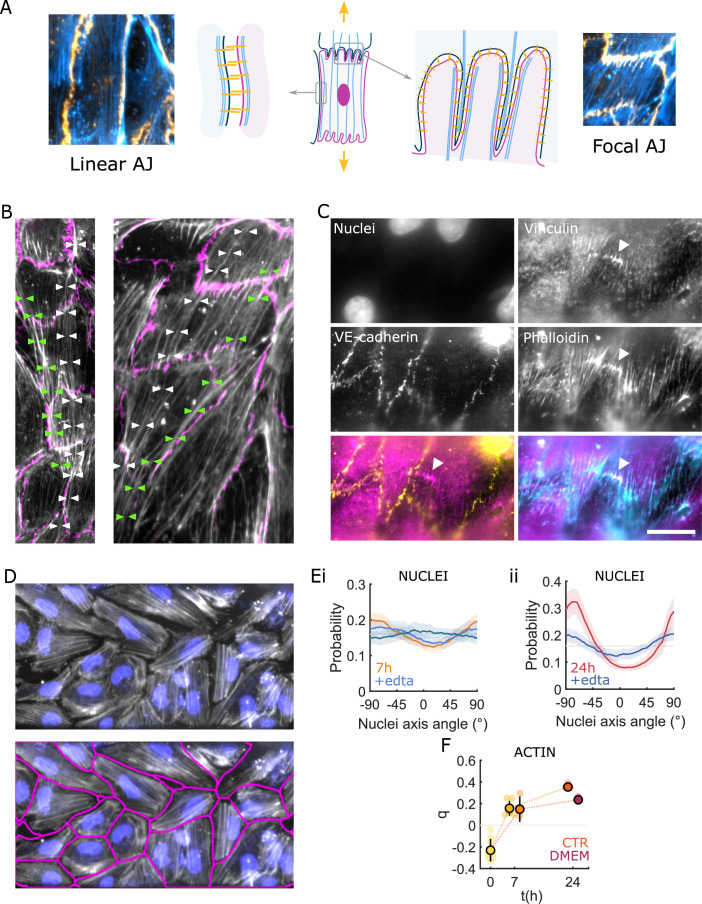
Cell-cell junctions and focal adhesions are necessary for actin alignment. **A** Schematics of the cell-cell junctions and associated actin fibers, showing a linear AJ (left) and a focal AJ (right), observed in monolayers under *ΔP*≈150Pa. **B** Endothelium stained for VE-cadherin (pink) and phalloidin (white) after 7h of stretch at *ΔP*≈650Pa, with long transcellular actin cables manually traced (white and green triangles). **C** Endothelium stained for nuclei, vinculin (magenta), VE-cadherin (yellow) and phalloidin (cyan) after 7h of stretch at *ΔP*≈650Pa, showing a line of clustered focal adhesion (white arrowhead) with actin fibers anchoring (cyan), away from cell-cell junctions (yellow). Scale bar 20 *μ*m. **D** EDTA-treated endothelium stained for nuclei (cyan) and phalloidin (white) after 7h of stretch at *ΔP*≈650Pa, with cell border outlined (magenta, bottom), showing elongated cells whose orientation follows stress fiber orientation. **E** Probability distribution of the nuclei long axis orientation at 7 h (**i**) and 24 h (**ii**) for control (orange, n=7 (7h); red, n=5 (24h)) and EDTA-treated in control medium (blue, n=5 (7h), n=6 (24h)) or DMEM (teal, n=3 (7h)) endothelia. **F** Nematic order parameter q as a function of time for EGM2-cultured (light orange, n=5 (7h); red, n=3 (24h)) and DMEM-cultured monolayers (dark orange, n=3 (7h); purple, n=2 (24h)), same data as in Fig. 2civ. Source data

Stress fibres have to be anchored to transmit forces, usually at FAs and AJs^40^. Here, in tissues under tension, two types of AJ are observed, namely, classical linear AJs and focal AJs (Fig. 3a and Extended Data Fig. 3a), known to form under tension^41,42^, which enable actin anchoring and long transcellular actin cables (Extended Data Fig. 3b). Focal AJs are found mostly at longitudinal cell–cell interfaces (Fig. 3a), whereas linear AJs with parallel stress fibres are found mostly at circumferential interfaces (Extended Data Fig. 3a), confirming that tension needs to be orthogonal to the interface to trigger focal AJ formation. Interestingly, the stress fibres in focal AJs are spaced regularly (Fig. 3a), suggestive of an optimization of the mechanical load distribution. A similar distribution is seen in anchoring at FAs, with FAs clustered together along a line, away from the cell periphery (Extended Data Fig. 3c). The lines along which FAs accumulate appear orthogonal to the orientation of stress fibres, and both stress fibres and FAs appear to be regularly spaced (Fig. 3b).

To investigate possible tension sensing by AJs, we stained for vinculin, a known mechanosensor and actin regulator, which has been previously shown to be recruited to focal AJs under tension. Vinculin co-localizes with focal AJs but also with linear AJs (Fig. 3c,f), contrary to what has been previously reported^42^. In our system, linear AJs can be subjected to tension parallel to their axis. This is consistent with the hypothesis that vinculin is recruited by high tension in junctions^42,43^, but suggests that this effect can occur without the remodelling into focal AJs.

To validate the putative role of junctions and FAs as mechanosensory hubs regulating actin, we first treated the monolayers before and during the pressure increase with EDTA to perturb AJs and maintain the presence of FAs (Fig. 3d). The actin network in EDTA-treated tissues after stretch showed a weaker realignment (Fig. 3d,e), indicating that AJs are involved in actin reorientation and/or tension sensing. EDTA-treated tissues continue to exhibit FAs, as shown by the vinculin dots (Fig. 3d), which might be responsible for the weaker sensing. To probe the role of FAs, we used endothelial tissues cultured with Dulbecco’s modified Eagle’s medium (DMEM), which have fewer and smaller FAs (Fig. 3f). These tissues show an intact response (Fig. 3f and Extended Data Fig. 3f). When further treated with EDTA, they exhibit a complete loss of actin fibre circumferential orientation at 7 h (Fig. 3g,e). In the DMEM + EDTA treatment, individual cells still possess ordered stress fibres that show some remodelling, with the formation of thick bundles (Fig. 3g), consistent with a possible role for the remaining small FAs in tension sensing. Neither cells nor nuclei are collectively aligned in the tension direction (Fig. 3g,e and Extended Data Fig. 3e). Cells are elongated in the direction of their internal stress fibres (Extended Data Fig. 3d), confirming that cell elongation is an active mechanism driven by actin. Taken together, these results suggest that AJs are sufficient for tension sensing and actin reorientation, and that FAs can partially rescue the mechanosensing when AJs are perturbed with EDTA.

### A model for tissue mechanics and actin nematodynamics

**Fig. 4:**
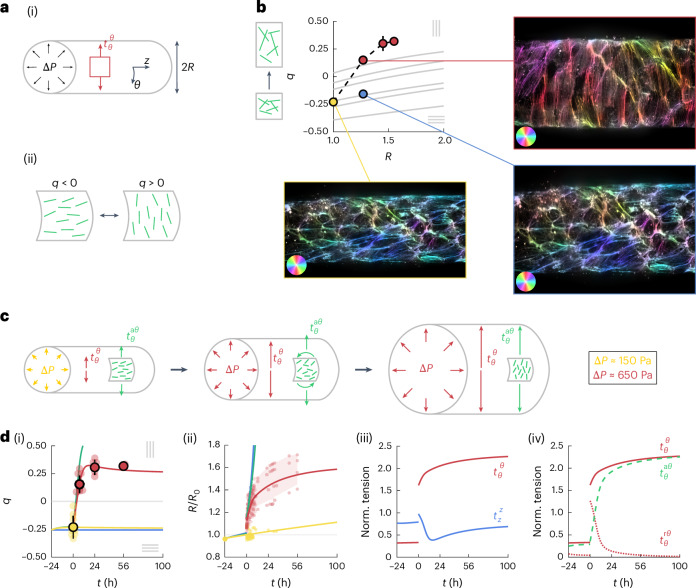
A model for tissue mechanics and actin nematodynamics recapitulates the response of endothelial tubes. **a**, Schematic of cylindrical tube or radius *R* subjected to the pressure difference Δ*P*, balanced by the circumferential tension ${t}_{\theta }^{\theta }$ (i). The change in orientation of actin fibres from longitudinal to circumferential corresponds to a change in sign of the order parameter *q* (ii). **b**, Circumferential actin nematic order *q* as a function of the normalized tube radius *R*/*R*_0_. Dots: experimental data, corresponding to **d**(i),(ii). Grey lines: numerically computed contribution of deformation by the tissue shear, starting with six sample images at *R*/*R*_0_ = 1. Insets: actin fibres colour coded based on their orientation, before tube stretching (yellow), after 7 h of 650 Pa pressure application (red) and for an artificial deformation of the initial image by an amount corresponding to the observed deformation *R*/*R*_0_ at 7 h (blue). **c**, Schematic of tube expansion dynamics and nematic reorientation induced by tube expansion. A sudden increase in the luminal pressure from Δ*P* ≈ 150 Pa to Δ*P* ≈ 650 Pa results in an instantaneous deformation, followed by a reorientation of actin fibres and an increase in the tension generated in actin stress fibres, ${{t}^{{\rm{a}}}}_{\theta }^{\theta }$, that slows down tube expansion. **d**, Actin order parameter *q* (i) and normalized tube radius *R*/*R*_0_ (ii) as a function of time, comparing the experimental data (dots) and model prediction (solid lines), for a constant pressure Δ*P* ≈ 150 Pa (yellow) and with pressure increase Δ*P* ≈ 650 Pa (red); the experimental data is as in Fig. 2a (with radius normalized by *R*_0_ for each experiment) and Fig. 2c(iv). Model predictions without the elastic component of the actin tension (green line, *K*_a_ = 0) and without the tension-coupling-inducing actin reorientation (blue line, *β* = 0) are also shown. Normalized total circumferential tension ${t}_{\theta }^{\theta }/{\zeta }_{0}$ (solid red line) and total longitudinal tension ${t}_{z}^{z}/{\zeta }_{0}$ (solid blue line) as a function of time (iii). Normalized total circumferential tension ${t}_{\theta }^{\theta }/{\zeta }_{0}$ (solid red line), circumferential tension in the actin stress fibre network ${{t}^{{\rm{a}}}}_{\theta }^{\theta }/{\zeta }_{0}$ (dashed green line) and residual tension ${{t}^{{\rm{r}}}}_{\theta }^{\theta }/{\zeta }_{0}$ (dotted red line) (iv). Source data

To test our hypothesis linking actin dynamics to tissue mechanics, we developed an active surface description of the endothelial tube (Fig. 4a).

#### Actin nematodynamics

We first investigated actin reorientation dynamics during tube expansion. We asked if the reorientation of actin stress fibres was a direct consequence of the anisotropic deformation induced by the circumferential elongation of the tube (Fig. 4b). However, we found that actin stress fibres are not simply following tube deformation (Fig. 4b and Supplementary Section 1.1.2).

We then asked if the *q* dynamics could be explained by a generic nematodynamics description (Fig. 4c). On the application of additional pressure, *q* switches sign but eventually recovers a similar magnitude than before pressure application, suggesting that the net effect of pressure application is for actin stress fibres to maintain a similar level of organization as they strongly reorient. Therefore, we assumed that the actin fibres form a network in the nematic phase. We then write the following equation for the nematic tensor *q*_*ij*_:

1 $${D}_{t}{q}_{ij}=-\gamma \left(\frac{1}{2}{q}_{kl}{q}^{kl}-{q}_{0}^{2}\right){q}_{ij}+\beta {\tilde{t}}_{ij}^{{\,}\mathrm{r}}.$$

Here *D*_*t*_ is the co-rotational time derivative (Supplementary Section 1.1.2), 1/*γ* is the timescale of relaxation of the order parameter *q*_*ij*_, *q*_0_ is the magnitude of nematic order, *β* is a mechanosensitive coupling term between the actin order and ${\tilde{t}}_{ij}^{{\,}{\rm{r}}}$ is the traceless part of the residual tension ${t}_{ij}^{{\rm{r}}}$, defined in equations (3)–(5). We envision that this residual tension corresponds to the tension supported by cell–cell junctions. In such a picture, anisotropic junctional tension, giving rise to the coarse-grained tissue-level residual tension ${t}_{ij}^{{\rm{r}}}$, could trigger differential actin network assembly and anchorage to the membrane, leading to anisotropic remodelling of actin stress fibres. Indeed, cell–cell junctions appear to be differently organized depending on their direction relative to the direction of tissue stretch (Extended Data Fig. 3) and actin reorientation relies on intact cell–cell junctions (Fig. 3).

#### Active viscoelastic model of tissue tension

The luminal pressure in the tube Δ*P*, its radius *R* and circumferential tension ${t}_{\theta }^{\theta }$ are related by Laplace’s law:

2 $$\Delta PR={t}_{\theta }^{\theta },$$

such that at constant pressure and for an increasing tube radius, the circumferential tension must increase. To account for this increase, we assumed that the tissue tension *t*_*ij*_ stems from an elastic tension from the actomyosin network ${t}_{ij}^{\rm{a}}$, acting along actin stress fibres, and a residual viscoelastic tension from the other tissue components ${t}_{ij}^{\rm{r}}$ such that

3 $${t}_{ij}={t}_{ij}^{\,\rm{a}}+{t}_{ij}^{\rm{r}},$$

4 $${t}_{ij}^{\,\rm{a}}=({\zeta }_{0}+{K}_{{\rm{a}}}s){n}_{ij},{D}_{{t}}s={v}_{kl}{n}^{kl},$$

5 $$(1+\tau {D}_{{t}}){t}_{ij}^{\rm{r}}=\mu {v}_{ij}.$$

The tension in the actin network is taken to be proportional to the mean orientation tensor *n*_*ij*_ = *g*_*ij*_/2 + *q*_*ij*_, with *g*_*ij*_ being the surface metric tensor. Our rationale is that the tension in the actin network is generated by a set of actin fibres under tension (Fig. 4c). The actin network tension magnitude has a constant active contribution *ζ*_0_ and an additional contribution proportional to the elongational strain of actin stress fibres *s*, with *K*_a_ being a two-dimensional elastic modulus. The dynamics of elongational strain depends on the tissue shear *v*_*ij*_. Laser ablation experiments indicate that the tension is not simply acting along actin stress fibres, as the circumferential tension is larger than the longitudinal tension following pressure application, before actin fibres have reoriented (Figs. 1b(ii) and 2c and Extended Data Fig. 1a). This observation is consistent with a contribution to the total tension of the additional residual tension ${t}_{ij}^{\rm{r}}$, which we simply describe by a viscoelastic Maxwell model, with *μ* being the viscosity and *τ* being the viscoelastic relaxation timescale (equation (5)). At short timescales, the response of the material is that of a linear elastic material, in line with the linear deformation observed at high collagen concentration in the range of pressures considered here (Fig. 1c(i)).

#### Dynamics of tube expansion and fibre reorientation

**Extended Data Fig. 4:**
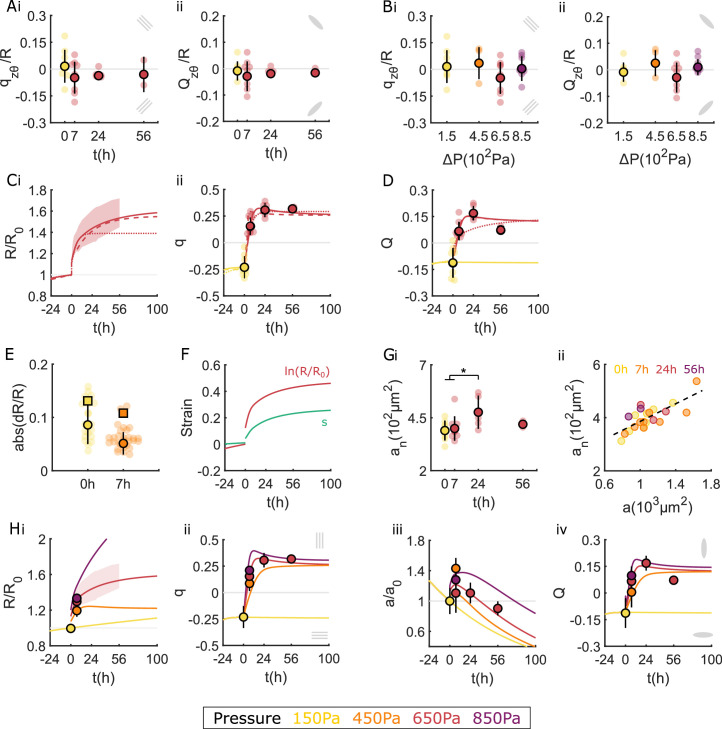
A model for tissue mechanics and nematodynamics recapitulates the response of endothelial tubes. **A,B** Value of normalized off-diagonal component *q*_*zθ*_/*R* of actin orientation tensor (**Ai**,**Bi**) and *Q*_*zθ*_/*R* of cell elongation tensor (**Aii**,**Bii**) at different time after pressure increase from 150 Pa (yellow) to 650 Pa (red) (**A**) and 7 hours after different pressure increases (**B**). **Ai** n=8 (0 h), n=9 (7 h), n=5 (24 h), n=2 (56 h). **Aii** n=8 (0 h), n=7 (7 h), n=5 (24 h), n=2 (56 h). **Bi**) n=8 (150 Pa), n=4 (450 Pa), n=9 (650 Pa), n=7 (850 Pa). **Bii** n=8 (150 Pa), n=4 (450 Pa), n=7 (650 Pa), n=7 (850 Pa). **C** Time evolution of normalized radius *R*/*R*_0_ (**i**) and actin order parameter *q* (**ii**) for *γ* = 10.8 h^−1^ and *β* = 8 N^−1^.m.h^−1^, other parameters being the same as in optimal fit (dotted line) and for best fit with *γ* = 0 h^−1^ (small dotted line) compared to the optimal fit (solid line). The experimental data are the same as in Fig. 2a,civ. **D** Time evolution of cell elongation *Q* for *λ* = 0 (small dotted line) and *λ* = 30.2 h^−1^ (dotted line) with all other parameters being the same as in optimal fit, compared to the optimal fit result (solid line). The experimental data are the same as in Fig. 2div. **E** Absolute relative radius change when increasing pressure from 150 Pa to 650 Pa (yellow) and when decreasing pressure back to 150 Pa 7 hours later (orange), comparing the experimental data (dots, same data as in Fig. 2bi) to the theoretical prediction (squares, Supplementary Section 2.5). **F** Tissue strain $\ln (R/{R}_{0})$ (red) and actin strain *s* (green) as a function of time, in the model. **Gi** Mean nucleus area as a function of time (experimental data). **Gii** Nucleus area as a function of cell area after 0 (yellow), 7 (orange), 24 (red) and 56 (purple) hours, showing a linear correlation. Data are the same as in Fig. 5aii, Extended Data Fig. 4Gi. **H** Time evolution of normalized radius *R*/*R*_0_ (**i**), actin order parameter *q* (**ii**), mean cell area (**iii**) and cell elongation (**iv**) for *ΔP*≈150Pa (yellow, n=8), 450Pa (orange, n=7), 650Pa (red, n=3) and 850Pa (purple, n=2) (lines: model, same curve as in Figs. 4di-ii, 5aii,5bii for *ΔP*≈150 and 650Pa, dots: experiment, same as in Fig. 5c). Source data

We found that equations (1)–(4) account for the dynamics of the actin nematic order (Fig. 4d(i)). The increase in circumferential tension after the application of additional tube pressure leads to the reorientation of actin stress fibres from longitudinal to circumferential on a timescale of ~1 h (*γ* = 1.3 ± 1.3 h^−1^; Supplementary Section 1.2 provides details for of mean and standard deviation of uncertainty analysis for this and other parameters). This timescale is comparable in magnitude to previously reported values of actin reorientation in endothelial cells under cyclic stretch^20,44–46^. We also note that in line with the model prediction, we did not observe notable deviation of the fibre orientations from the longitudinal or circumferential direction (Extended Data Fig. 4a,b).

**Extended Data Fig. 5:**
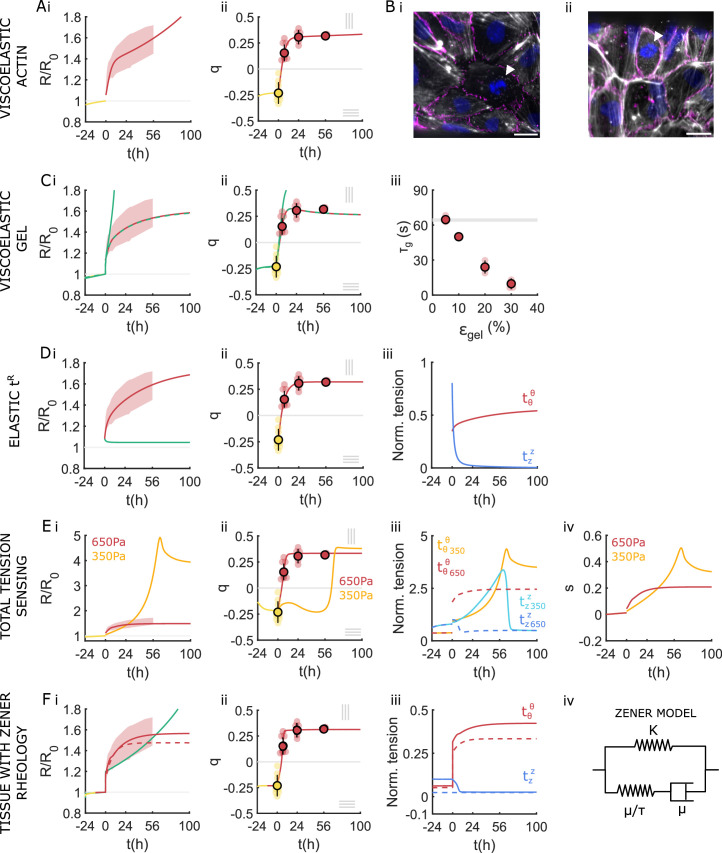
Exploring alternative models of endothelial tube mechanics. **A** Time evolution of normalized radius *R*/*R*_0_ (**i**) and actin order parameter *q* (**ii**), in a model with viscoelastic actin accounting for actin strain memory loss (solid line, Supplementary Section 1.3.2) compared to experimental data (shadow, dots, same data as in Fig. 2a,civ). **B** Endothelium stained for DAPI (blue), VE-cadherin (magenta) and phalloidin (white) under low pressure (**i**) and high pressure (**ii**), showing a mitotic cell (arrowhead) with condensed chromatin and no stress fibers. Scale bar 20 *μ*m. **C** Time evolution of normalized radius *R*/*R*_0_ (**i**) and actin order parameter *q* (**ii**) taking into account the contribution of the surrounding gel (green lines, Supplementary Section 1.3.3) or not (red lines, same data as in Fig. 4di,ii), removing actin elasticity (continuous lines) or not (dashed lines), based on the values for the relaxation time constant *τ*_g_ (**iii**) obtained by fitting with an exponential the time evolution of the stress after a strain step of varying amplitude (Extended Data Fig. 1Bi). **D** Time evolution of normalized radius *R*/*R*_0_ (**i**) obtained for a model where the total tension is the sum of a purely elastic tension $${t}_{ij}^{\rm{r}}$$ and a tension $${t}_{ij}^{\rm{a}}$$ oriented along actin stress fibers, either purely elastic (green line, Supplementary Section 1.3.4) or viscoelastic (red line, Supplementary Section 1.3.4), fitted to experimental data. Red shadow indicates experimental data as in Fig. 2a. Here the actin stress fibers order parameter has been fitted by $$q(t)=-0.25+0.57$$$$\times (1-\exp (-0.17t))$$ (**ii**, dots show experimental data as in Fig. 2civ). **Ciii** Time evolution of normalized circumferential tension $${t}_{\theta }^{\theta }/K$$ (red) and longitudinal tension $${t}_{z}^{z}/K$$ (blue), computed from the model in (i), with viscoelastic actin oriented tension. **E** Time evolution of normalized radius *R*/*R*_0_ (**i**) and actin order parameter *q* (**ii**) after a pressure increase from 150 Pa to 350 Pa (orange) and 650 Pa (red), obtained from a model where actin fibers mean orientation dynamics is coupled to total tension (solid lines, Supplementary Section 1.3.6), fitted to experimental data. Shadow and dots indicate experimental data as in Fig. 2,civ. **Eiii** Time evolution of normalized circumferential tension $${t}_{\theta }^{\theta }/{\zeta }_{0}$$ (red and orange) and longitudinal tension $${t}_{z}^{z}/{\zeta }_{0}$$ (blue and cyan) for a pressure increase from 150 Pa to 350 Pa (red and blue) and 650 Pa (orange and cyan), computed from the model in (i). **Eiv** Time evolution of actin strain *s* after a pressure increase from 150 Pa to 350 Pa (orange) and 650 Pa (red), computed from this model. **Fi** Time evolution of normalized radius *R*/*R*_0_ obtained from the following models: tissue described as an isotropic material following a Zener rheology, without (green, Supplementary Section 1.3.7), or with isotropic active tension (red dashed line, Supplementary Section 1.3.7), and tissue described as an elastic material with Zener rheology, together with an active tension contribution oriented along actin stress fibers (red solid line, Supplementary Section 1.3.8). Shadow indicates experimental data as in Fig. 2a. **Fii** Time evolution of actin order parameter *q* obtained from the model describing the tissue as an elastic material with Zener rheology together with actin oriented active tension (red), fitted to experimental data (dots, same data as in Fig. 2civ). **Fiii** Time evolution of normalized circumferential tension $${t}_{\theta }^{\theta }/K$$ (red) and longitudinal tension $${t}_{z}^{z}/K$$ (blue), computed from either a model describing the tissue as an elastic material with Zener rheology and subjected to actin oriented active tension (solid line) or a model describing the tissue as an isotropic material following a Zener rheology, together with isotropic active tension (dashed lines). **Fiv** Schematics of the Zener rheology, consisting of a spring of elastic modulus *K*, in parallel with a serial association of a dashpot of viscosity *μ* and a spring of elastic modulus *μ*/*τ*, with *τ* a characteristic time. Source data

The model also accounts quantitatively for the dynamics of tube expansion (Fig. 4d(ii)). In the model, the initial jump in tube radius is limited by the initial elastic response of the tissue (Fig. 4c,d(iii) and Extended Data Fig. 4e). The order of magnitude of the fitted values of the elastic coefficients, *K*_a_ = 0.22 N m^–1^ (0.22 ± 0.03 N m^–1^) and *K* = *μ*/*τ* = 0.29 N m^–1^ (0.31 ± 0.03 N m^–1^), match well with values extracted from pressure ramp application, of the order of 0.26–0.4 N m^–1^ (Fig. 1c). Elastic residual tension in the tissue then relaxes, leading to further tube expansion (Fig. 4c,d(ii),(iv)). The reorientation and strain of elastic fibres, however, allows limiting and eventually fully opposing expansion of the tube (Fig. 4d(i),(ii) (blue and green curves) and Extended Data Fig. 4f). Alternative models did not account as well for the experimental data, except for a model with a mechanosensitive coupling of the nematic order to the full tension tensor *t*_*ij*_ (Supplementary Section 1.3.6 and Extended Data Fig. 5e), and a model with active tension along the actin fibres, but where the long-timescale elastic response does not arise from actin fibres but from the rest of the tissue (Supplementary Section 1.3.8 and Extended Data Fig. 5f). The total tension values (Fig. 4d(iii)) are anisotropic with a larger circumferential tension after pressure increase, in qualitative agreement with laser ablation experiments (Fig. 1b(ii) and Extended Data Fig. 1a). The model also predicts a larger longitudinal tension before pressure application (Fig. 4d(iii)), detected by laser ablation only on soft collagen (Fig. 1b(ii) and Extended Data Fig. 1a), possibly due to the weak longitudinal anisotropy of the high-density monolayers used for these experiments.

Overall, we propose that actin stress fibres reorient along the direction of highest residual tissue tension, allowing the tissue to resist anisotropic deformation (Fig. 4d(i),(ii)).

#### Dynamics of cell area

**Fig. 5:**
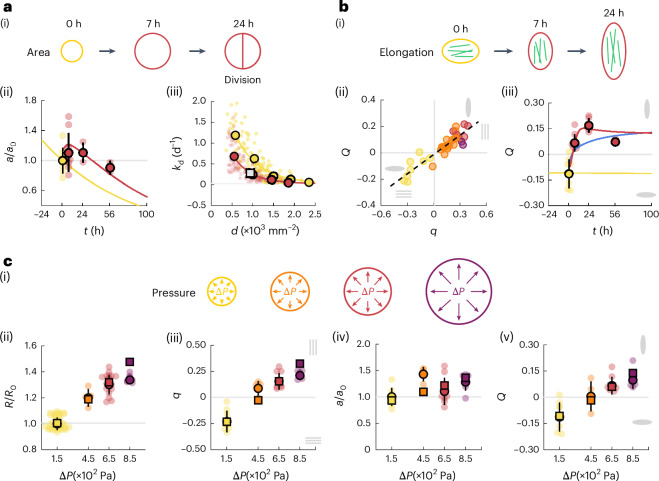
Dynamics of cell area, elongation and response to a range of pressures. **a**, Schematic of the mean cell area dynamics (i). Normalized cell area as a function of time (ii), comparing the experimental data (dots) and model prediction (solid lines), for a constant pressure Δ*P* ≈ 150 Pa (yellow, *n* = 8) and after the pressure increase Δ*P* ≈ 650 Pa (red, *n* = 7 (7 h), *n* = 3 (24 h) and *n* = 2 (56 h)). Proliferation rate *k*_d_ as a function of cell density (iii), measured between *t* = 0 and *t* = 7 h, for monolayers under low pressure Δ*P*_0_ ≈ 150 Pa (yellow dots) and high pressure Δ*P*_m_ ≈ 650 Pa (red dots). Lines: exponential fit. Grey square: prediction from isotropic shear decomposition. **b**, Schematic of the cell elongation dynamics (i). Cell circumferential elongation *Q* as a function of the actin nematic order parameter *q* (ii), showing a linear empirical correlation, colour coded for time (0 h, yellow; 7 h, orange; 24 h, red; 56 h, purple), with the experimental data as in Fig. 2d(iv). Cell circumferential elongation *Q* as a function of time (iii), comparing the experimental data (dots) and model prediction (solid lines), for a constant pressure Δ*P* ≈ 150 Pa (yellow, *n* = 8) and with a pressure increase Δ*P* ≈ 650 Pa (red, *n* = 7 (7 h), *n* = 3 (24 h) and *n* = 2 (56 h)), with the experimental data as in Fig. 2c(iv),d(iv). Blue line: model prediction for the case in which the cell elongation follows tissue deformation. **c**, Schematic of the different pressures applied to the endothelial tube (i). Normalized tube radius *R*/*R*_0_ (ii), actin nematic order parameter *q* (iii), cell area *a*/*a*_0_ (iv) and cell elongation *Q* (v) as a function of pressure, measured 7 h after pressure step application, comparing the experimental data (circles) and model prediction (squares). (ii) *n* = 30 (150 Pa), *n* = 4 (450 Pa), *n* = 18 (650 Pa) and *n* = 7 (850 Pa). (iii) and (iv) *n* = 8 (150 Pa), *n* = 4 (450 Pa), *n* = 9 (650 Pa) and *n* = 7 (850 Pa). (v) *n* = 8 (150 Pa), *n* = 4 (450 Pa), *n* = 7 (650 Pa) and *n* = 7 (850 Pa). Source data

We then investigated the dynamics of the cell area, which increases transiently after pressure application before decreasing over 56 h (Fig. 5a). The nuclei area mirrors this trend, suggesting that nuclei are also stretched transiently (Extended Data Fig. 4g). In the absence of cell apoptosis, the cell area *a* follows an isotropic shear decomposition^47,48^ (Fig. 4e(i)):

6 $$\frac{1}{a}\frac{{\rm{d}}a}{{\rm{d}}t}=\frac{1}{R}\frac{{\rm{d}}R}{{\rm{d}}t}-{k}_{{\rm{d}}}.$$

This relationship indeed accounts well for the observed dynamics of the cell area, for a constant cell division rate *k*_d_ = 0.27 ± 0.07 d^−1^ (Fig. 5a(ii)). After pressure increase, the cell area increases due to fast tissue expansion, and then relaxes due to continued cell division (Fig. 5a(i),(ii)).

We then directly measured the cell division rate under both 150 Pa and 650 Pa pressures with a proliferation assay (Fig. 5a(iii)). To avoid confounding effects of changes in cell density brought by tissue expansion, we measured the cell division rates in endothelial tubes seeded with different initial cell densities. The cell division rate sharply decreases with cell density, with a dependency well fitted by an exponential function (Fig. 5a(iii)). One may then expect that increasing tissue tension leads to larger cell area and, therefore, higher proliferation. Surprisingly, however, the cell division rate is lower for larger pressure, implying that increased tissue tension slows down proliferation (Fig. 5a(iii)). Therefore, tissue tension influences cell proliferation beyond changing the cell area. The predicted cell division rate obtained from isotropic shear analysis (Fig. 5a(ii)) matches very well with its measured value under high pressure, at the average density of the experiment (Fig. 5a(iii)), consistent with cell proliferation being responsible for the decrease in cell area after 7 h and the absence of apoptosis.

#### Dynamics of cell elongation

We then asked if the dynamics of cell elongation could be understood with an anisotropic shear decomposition, where the average cell elongation changes due to tissue anisotropic shear and cellular rearrangements^47,48^. We postulated that cellular rearrangements are driven by the difference between the actin nematic order and the cell elongation, based on the observed empirical linear correlation between *Q* and *q* (Fig. 5b(i),(ii)) and on the absence of cell elongation when actin is depolymerized (Fig. 2f), leading to the following dynamics:

7 $${D}_{t}{Q}_{ij}={\tilde{v}}_{ij}-\lambda ({Q}_{ij}-\alpha {q}_{ij}),$$

where ${\tilde{v}}_{ij}$ is the traceless part of the tissue shear *v*_*ij*_ and 1/*λ* is a relaxation timescale of the cell elongation *Q*_*ij*_ to a preferred value *αq*_*ij*_. This hypothesis accounts well for the dynamics of cell elongation (Fig. 5b(iii)) for a timescale 1/*λ* ≈ 45 min (1 ± 1.1 h), indicating that cell elongation is largely slaved to the orientation of actin fibres over the experiment duration. Indeed, large values of *λ*, corresponding to faster relaxation to the actin fibre nematic order, can still account for the elongation dynamics (Extended Data Fig. 4d). The final decrease in cell elongation at 56 h, however, is not predicted by the theory and could arise from cell divisions that are oriented circumferentially (Fig. 2e), indicating a possible decoupling between the actin stress fibres and cell elongation nematics on longer timescales.

#### Effect of pressure magnitude

To further verify that the proposed physical description indeed accounts for endothelial tube dynamics under pressure, we obtained the predicted tube radius *R*, actin order *q*, cell elongation order *Q* and the cell area *a* for a range of pressures. The model predicts that for larger pressures, both tube radius and mean cell area expands more and that actin fibres and cell elongation reorient more strongly along the circumferential direction (Fig. 5c and Extended Data Fig. 4h). To test these predictions, we measured the response of the tissue at 7 h for different imposed pressures (Δ*P* ≈ 450 Pa and Δ*P* ≈ 850 Pa). The tube radius, mean cell area, actin orientation and cell orientation at 7 h scale roughly linearly with the applied pressure, in good agreement with the theoretical predictions (Fig. 5c and Extended Data Fig. 4h). Overall, we conclude that actin fibre orientation is sensitive to the physiologically relevant range of pressure variations from 0 Pa to 850 Pa, owing to the mechanosensitivity of the nematic order of actin stress fibres, encoded by the parameter *β* in equation (1).

## Discussion

Here we demonstrate that pressure application on a reconstituted endothelial tube leads to actin stress fibre circumferential alignment, parallel to the direction of applied stretch and maximum tension. This response is consistent with the alignment of fibres along the direction of maximum tension reported in the epithelia in vivo^49^, but contrasts with actin fibres in endothelial cells reorienting perpendicularly to the direction of stretch under the imposed cyclic deformation^5^.

Tubes under tension famously tend to be unstable^50,51^. Indeed, Laplace’s law (equation (2)) implies that the circumferential tension must increase in a pressurized expanding tube. This indicates that the endothelial tube should respond elastically to resist expansion. However, the average cell area and circumferential cell elongation decrease. This led us to propose that the endothelial tube expansion is instead limited by actin fibre reorientation and elongational strain. In addition to the actin stress fibre nematics, we have simultaneously examined the dynamics of cell elongation. Although these two nematic fields have been proposed to be decoupled in epithelial layers^52^, here we find that they follow each other for most of the tube expansion.

Our microstretcher allows to set a fixed value of pressure magnitude, thereby preventing tension relaxation in the tissue. It mimics a key in vivo scenario, modelling pressure increases due to the onset of heart beat or hypertension, with physiologically relevant pressure differences^3,38,39^. The cellular dynamics we report here could underlie vessel remodelling and pathological mechanoadaptation of vessels. Tension-induced remodelling of AJs is evident by shape change and actin and vinculin association, a mechanism previously linked to the protection of tissue integrity and barrier function^41,42,53,54^. In vivo, an increase in luminal pressure has been reported to trigger the radial growth of vessels and junction remodelling along the tension direction in zebrafish, mirroring our observations^55^.

Pressure-induced remodelling is believed to underlie other fluid-transporting tubular networks, such as mammary glands, bronchial tree or lymphatic network, potentially presenting a mechanism extending beyond the cardiovascular network^56,57^. Therefore, luminal-pressure-induced wall tension may serve as a universal regulator in vessels in vivo, governing the adaptation of their properties and shape and optimizing them for their specific function. By linking the behaviour of biological components to the emergent tissue mechanics, we present a key step to understand the process of mechanoadaptation in vessels. It would be interesting in further studies to use our microstretcher setup to further dissect the role of cytoskeletal components and different types of endothelial cell in endothelial tube mechanics to study the role of chirality, which was recently reported in microvessels both in vivo and in vitro^58^, or to apply oscillatory pressures to mimic the pulsatility of the blood flow. In addition, interactions with other cell types such as smooth muscle cells and fibroblasts could play a role in mechanoadaptation in larger vessels.

## Methods

### Microvessel-on-chip fabrication

The microvessel-on-chip system consists of a chamber that houses a 120 μm diameter endothelium-lined channel embedded in a soft collagen hydrogel^25^.

After fabricating the polydimethylsiloxane (PDMS) housing with its inlet and outlet ports, a 120 μm diameter acupuncture needle (Seirin) was introduced into the chamber and the housing was bound to a coverslip through plasma activation. Two PDMS reservoirs were sealed with liquid PDMS to the inlet and outlet ports, their inner diameter matching the diameter of standard plastic straws. The chamber was sterilized with 70% ethanol and 20 min of UV light exposure. To improve collagen adhesion to the PDMS walls, the chamber was coated with 1% polyethylenimine (an attachment promoter; Sigma-Aldrich) for 10 min followed by 0.1% glutaraldehyde (a collagen crosslinker; Polysciences) for 20 min. Collagen I was isolated from the rat tail tendon, as described previously^59^, to obtain a stock solution of 12 mg ml^−1^. Type I collagen solution was then prepared by diluting the acid collagen solution in a neutralizing buffer at a 1:1 ratio, pipetted into the housing chamber and allowed to polymerize in a tissue culture incubator for 15 min for the baseline collagen concentration of 6 mg ml^−1^ and for up to 4 h for lower collagen concentrations. The acupuncture needle was then carefully removed, and the needle holes were sealed with vacuum grease (Bluestar Silicones) to avoid leakage.

### Cell culture, seeding and inhibition

Human umbilical vein endothelial cells (Lonza) were cultured using standard protocols in an endothelial growth medium (EGM2; Lonza) and used up to passage seven. The medium was changed every other day and cells were passed on confluence, on average every 4 days. For channel seeding, on confluence, endothelial cells were detached from the flask using trypsin (Gibco and Life Technologies) and concentrated to 10^7^ cells ml^–1^. Then, 1 μl of the concentrated cell suspension was pipetted through the inlet port of the device. After 5 min incubation, non-adhering cells were gently flushed out. After 1 h, a flow rate of 2 μl min^−1^ was applied via a syringe pump (PHD ULTRA, Harvard Apparatus). A confluent monolayer was obtained in 24 h. For the pharmacological experiments, cells were cultured for 1 h before the experiment in the presence of 100 nmol l^–1^ cytochalasin D in EGM2 for actin disruption, and 5 mmol l^–1^ EDTA in EGM2 or 2 mmol l^–1^ EDTA in DMEM for cell–cell junction perturbation.

### Monolayer stretch

**Extended Data Fig. 6:**
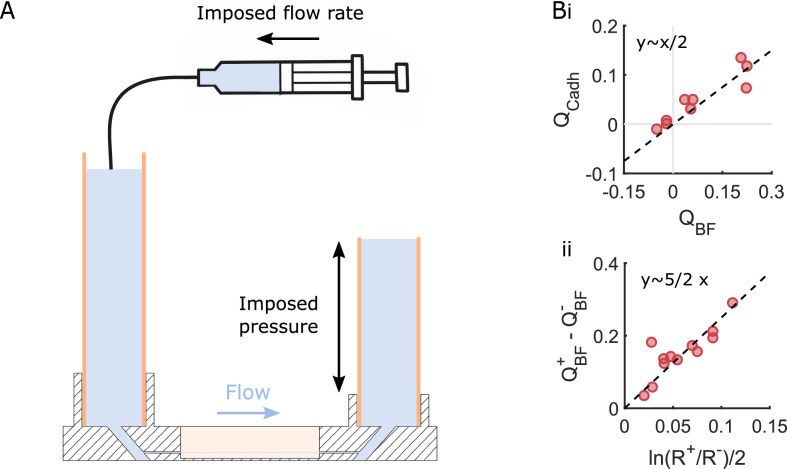
Schematics of the system and determination of the coefficient in the cell elongation nematic parameter Q. **A** Schematics of the microfluidic set up showing the imposed flow rate at the channel inlet and the imposed pressure at the channel outlet. **Bi** Cell order parameter extracted from immunostaining of VE-cadherin *Q*_Cadh_ as a function of the cell order parameter extracted from phase contrast brightfield *Q*_BF_ showing a linear correlation with a 0.5 coefficient. **Bii** Jump in the cell order parameter, extracted from phase contrast brightfield *Q*_BF_ as a function of the logarithm of the jump in radius, showing a linear correlation with a 5/2 coefficient. ${Q}_{\rm{BF}}^{+}$ and ${Q}_{\rm{BF}}^{-}$ (resp. *R*^+^ and *R*^−^) are respectively the value of *Q*_BF_ (resp. *R*) just before and just after the pressure increase. Source data

A hydrostatic pressure head was used to impose both luminal pressure as the flow rate was imposed using a syringe pump (PHD ULTRA, Harvard Apparatus). The hydrostatic pressure head was created by the medium filling a plastic straw of a specific length attached to the channel outlet (Extended Data Fig. 6a). A PDMS base was created by puncturing a 5 mm diameter hole in a PDMS cubic block (1 cm on a side) and gluing the block to the inlet and outlet. The straws were then inserted into the PDMS base. The inlet straw was continuously replenished using the syringe pump at a fixed flow rate. The pressure within the channel is, therefore, set by the height of the outlet straw, whereas the luminal flow rate (and the ensuing pressure gradient) is set by the syringe pump flow rate. The height of the medium in the inlet straw is equal to that in the outlet straw height plus the pressure gradient due to the luminal flow and the channel hydraulic resistance.

During monolayer growth, the flow rate was set to 2 μl min^−1^ and the outlet pressure to 100 Pa, which was maintained for the control channels. A pressure gradient of 100 Pa is established between the inlet and outlet. For the stretch experiments, at *t* = 0, both inlet and outlet reservoirs were filled with a syringe in less than a minute to impose a hydrostatic pressure at the outlet of 400 Pa, 600 Pa or 800 Pa, defined by the height of the reservoir. The outlet pressure was maintained constant for the duration of the experiments (7 h, 24 h or 56 h), whereas the inlet pressure was maintained by the flow rate imposed with a syringe pump. The flow rate was increased during the course of the experiments, to 3 μl min^−1^ at *t* = 0 h and to 4 μl min^−1^ after 24 h, to account for the increased diameter and to maintain the pressure gradient roughly constant—100 Pa. We neglected the effect of the pressure gradient, in both experimental quantifications and theoretical modelling, and the average pressure of the channel was considered to be around 150 Pa, 450 Pa, 650 Pa and 850 Pa for outlet pressures of 100 Pa, 400 Pa, 600 Pa and 800 Pa, respectively.

### Measurement of channel deformation

An increase in the luminal pressure leads to a pressure difference across the vessel wall and subsequent channel dilation and circumferential stretch (Supplementary Video 1). The circumferential strain, defined as the ratio of the increase in perimeter of the cross-section of the channel to the initial perimeter, was obtained from the vessel diameter.

Channel diameters were automatically measured, as previously described^32^. Briefly, channel edges are detected by identifying the position of the peaks in the intensity gradient along the vertical direction. The diameter is then the mean distance between the two peaks.

### Laser ablation

Laser ablation experiments were performed as described elsewhere^60,61^. The chip was placed on a TriM Scope II microscope (LaVision BioTec) equipped with a femtosecond Mai Tai HP DeepSee laser (Spectra-Physics), an Insight DeepSee (Spectra-Physics) laser and a XLPLN25XWMP2 (Olympus) ×25 water-immersion objective. LifeAct mCherry was excited through two-photon excitation using the InSight laser set to 1,160 nm and ablation was performed using the Mai Tai laser set to 820 nm and exit power at 0.45 mW. Using an electro-optic modulator, the region to be ablated was defined as an *x*–*y* region of interest of 4.5 × 76 μm^2^ located at the level of the actin cytoskeleton and oriented either longitudinally or circumferentially. Endothelial cells were imaged with a frame every 130 ms, for 5 time frames before ablation, then ablated for 2 frames and, finally, imaged for 53 time frames (Supplementary Video 2). The same channel served for six ablations, three in each direction and alternating, starting from 1.5 mm away from the channel border and separated by 1.5–2 mm to avoid the influence of one cut on the adjacent cuts. To compute the initial recoil velocity, data were analysed by manually measuring the distance travelled by the edge of the cut in the first frame relative to its initial position.

### Rheology measurement of collagen gel

Rheology measurements of the collagen gels were performed following a previously published protocol^30^. We measured stress relaxation using a stress-controlled rheometer (Anton Paar MCR 301) equipped with measuring plates of 25 mm in diameter with stainless steel surfaces. To prevent slipping of the collagen gel, a profiled, surfaced top plate was used. The collagen hydrogel with a concentration of 6 mg ml^–1^, prepared following the same protocol as the one used for the microvessel fabrication, was deposited on the bottom plate cooled at 4 °C. The top plate was lowered quickly, before the gelation of the collagen was initiated by heating the plate to 37 °C. Gelation was monitored with continuous oscillations at a strain rate of 0.01 and frequency of 1 rad s^–1^. The mechanical measurements were performed after the storage modulus reached a stable value. Different strains ranging from 5% to 30% were applied with a rise time of 0.1 s and then maintained for 5 min to observe stress relaxation. Young’s modulus *E*_g_ was extracted from the shear modulus *G* using the relationship *E*_g_ = 2*G*(1 + *ν*_g_), with *ν*_g_ = 0.3 (refs. ^62,63^).

### Measurement of monolayer stiffness

For instantaneous deformations of linear elastic materials, the relationship between tension and strain is dictated by Young’s modulus. We, therefore, used the present system to measure the Young’s modulus of the endothelium, as previously described^32^ (Supplementary Section 2).

#### Measurement of stress–strain relationship

Briefly, channels were subjected to a 1 min long pressure ramp, from 150 Pa to 1,000 Pa, and the circumferential strain was recorded (Supplementary Video 3). Young’s modulus is then extracted from the slope of the stress–strain curve, assuming that dissipative stresses are negligible. The pressure was increased at a constant speed by simultaneously filling the inlet and outlet reservoirs with a syringe pump at a 2 ml min^−1^ rate, which corresponds to 1,000 Pa min^−1^. Channels were imaged every second in phase contrast with a ×10 objective. The increase in diameter was then measured automatically using the same method as that for still snapshots (as mentioned earlier). A second method was used in which a similar 1 min long pressure ramp was applied, starting at 150 Pa but stopping at 650 Pa. Here there was no continuous imaging, a bright-field image was taken only at the beginning (150 Pa) and the end of the pressure application (650 Pa), from which the diameters were measured.

#### Fitting procedures for parameter inference

The stress–strain curves of control and pharmacologically perturbed monolayers on 2 mg ml^−1^ collagen, measured continuously, displayed a typical strain-stiffening behaviour (Fig. 1c,e). The cell monolayer was then modelled as a shell with a thickness of 3.6 ± 0.5 μm, composed of a nonlinear elastic Gent material; however, the hydrogel was modelled as a linear elastic material that decreases the pressure drop across the cell layer. The monolayer thickness was measured from the fluorescence images of the actin cytoskeleton obtained at the tube midplane. By fitting the model to the experimental curves, we inferred the Young’s modulus of the endothelium (Supplementary Section 2.2).

The stress–strain curves of the control monolayers on 6 mg ml^−1^ collagen, measured continuously, displayed a linear relationship (Fig. 1c) up to 900 Pa. The paired values of the control monolayers on 6 mg ml^−1^ collagen also showed a linear behaviour when connected to the reference state *D*_0_ = 125 μm at Δ*P* = 0 Pa. This reference state was determined using the Gent model (Supplementary Section 2.1.1) and consistent with a previous measurement of the bare channel diameter^25^. We, therefore, used the value of the radial strain at 650 Pa to estimate the Young’s modulus of these control monolayers (Fig. 1c,f). The paired values of the pharmacologically treated monolayers showed a strain-stiffening behaviour when connected to the reference state (Fig. 1f), consistent with the diameter at 650 Pa being above the threshold diameter for stiffening, estimated to be around 150 μm (Fig. 1e(i)). We, therefore, used the value of the radial strain at 150 Pa, still in the linear regime, to estimate the Young’s modulus of the cytochalasin-D- and EDTA-treated monolayers (Fig. 1f).

### Immunostaining

Cell–cell junctions were stained using a rabbit anti-VE-cadherin primary antibody (Abcam, 33168). Actin filaments and nuclei were stained using Alexa Fluor phalloidin (Invitrogen, Thermo Fisher Scientific, A12379) and DAPI (Invitrogen, Thermo Fisher Scientific D3571), respectively. In addition, a mouse anti-vinculin (Sigma-Aldrich, Merck V9264) and a mouse anti-vimentin (Abcam, 92547) primary antibodies were used to stain for FAs and intermediate filaments. Immunostaining was performed by the slow infusion of reagents into the microchannel. Cells were fixed in 4% paraformaldehyde (Thermo Fisher Scientific) for 15 min, rinsed with phosphate-buffered saline (PBS) and then permeabilized with 0.1% Triton in PBS for another 15 min. The channel was then perfused with a 3% bovine serum albumin solution in PBS for 1 h to block non-specific binding. Cells were incubated with the primary antibodies (1:400) in PBS for 1 h at room temperature and then rinsed with PBS for an additional 1 h. The channel was then perfused with the secondary antibodies (1:400), phalloidin (1:200) and DAPI (1:1,000,000) in PBS. Finally, the cells were incubated overnight in PBS at 4 °C. Samples were imaged using the NIS-Elements software (v. 5.02.03, build 1273) on an epifluorescence inverted microscope (Nikon ECLIPSE Ti) and/or a Crest X-Light confocal system mounted on an inverted microscope (Nikon ECLIPSE Ti).

### Analysis of orientation

For the statistical analysis of orientations, at least 15 images along the bottom half and the top half of the channel were acquired with a ×10 objective. A region of interest was then selected to match the area in focus. Angles were defined relative to the channel longitudinal axis, aligned to the horizontal axis. To be coherent with the tangential basis, we chose in the model (Fig. 4a(i)), angles were defined positive in the bottom half of a trigonometric circle and negative in its bottom half.

#### Actin and cell orientation

Actin fibre orientation was obtained from the images of phalloidin stainings using the OrientationJ plug-in in ImageJ^64^. The window size was set to 5 pixels and the cubic spline method was used. The probability distributions of the angles generated by the plug-in for each longitudinal position within one channel were then averaged together. The same pipeline was applied to the VE-cadherin immunostaining and to the bright-field images to estimate the cell elongation and orientation.

#### Nuclei orientation

Nuclei were segmented using a custom-made MATLAB (version R2021b) code. Each nucleus was fitted with an ellipse and the angle of the long axis of the ellipse was used as the nucleus angle. Angles were then binned to create a probability distribution.

#### Division orientation

Mitotic angles were manually measured from nuclear (DAPI) immunostainings. Dividing cells were identified by their condensed chromosomes and the angle between the segment connecting the two daughter nuclei and the longitudinal axis was measured using ImageJ. Angles were grouped and then binned to create a probability distribution.

#### Nematic order parameter calculation

The tensors *q*_*ij*_, *Q*_*ij*_ and ${{Q}_{\rm{n}}}_{ij}$ are defined as

8 $${q}_{ij}=\frac{1}{2}\left(\begin{array}{cc}-{R}^{2}\left\langle \cos 2{\theta }_{q}\right\rangle &R\left\langle \sin 2{\theta }_{q}\right\rangle \\ R\left\langle \sin 2{\theta }_{q}\right\rangle &\left\langle \cos 2{\theta }_{q}\right\rangle \end{array}\right),$$

9 $${Q}_{ij}=\frac{{\alpha }_{Q}}{2}\left(\begin{array}{cc}-{R}^{2}\left\langle \cos 2{\theta }_{Q}\right\rangle &R\left\langle \sin 2{\theta }_{Q}\right\rangle \\ R\left\langle \sin 2{\theta }_{Q}\right\rangle &\left\langle \cos 2{\theta }_{Q}\right\rangle \end{array}\right),$$

10 $${{Q}_{\rm{n}}}_{ij}=\frac{1}{2}\left(\begin{array}{cc}-{R}^{2}\left\langle \cos 2{\theta }_{\rm{n}}\right\rangle &R\left\langle \sin 2{\theta }_{\rm{n}}\right\rangle \\ R\left\langle \sin 2{\theta }_{\rm{n}}\right\rangle &\left\langle \cos 2{\theta }_{\rm{n}}\right\rangle \end{array}\right),$$

where *θ*_*q*_, *θ*_*Q*_ and *θ*_n_ are a set of angles obtained by the image analysis, as described above. In the case of the symmetric distribution of angles *θ*_*q*_, *θ*_*Q*_ and *θ*_n_ around 0, off-diagonal terms of the tensors *q*_*ij*_, *Q*_*ij*_ and ${{Q}_{\rm{n}}}_{ij}$ vanish and actin orientation, cell elongation and nucleus elongation can be described by the order parameters $q={q}_{\theta }^{\theta }=-\left\langle \cos 2{\theta }_{q}\right\rangle /2,Q={Q}_{\theta }^{\theta }=-{\alpha }_{Q}\left\langle \cos 2{\theta }_{Q}\right\rangle /2$ and ${Q}_{\rm{n}}=-{{Q}_{\rm{n}}}_{\theta }^{\theta }=\left\langle \cos 2{\theta }_{\rm{n}}\right\rangle /2$, respectively. We chose the convention in which a positive value of *q*, *Q* or *Q*_n_ indicates that the orientation is preferentially circumferential. Conversely, a negative value of *q*, *Q* or *Q*_n_ indicates a preferentially longitudinal orientation. The magnitude of *q*, *Q* or *Q*_n_ corresponds to the strength of the alignment along the circumferential or longitudinal direction.

#### Calculation of coefficient *α*_*Q*_

Because the orientation of junctions does not necessarily exactly reflect cell elongation, we introduce a correction factor *α*_*Q*_ as follows. For a uniform shear flow ${\tilde{v}}_{ij}$ (traceless part of the gradient of flow *v*_*ij*_) and in the absence of cellular rearrangements, we expect ${D}_{t}{Q}_{ij}={\tilde{v}}_{ij}$. In the context of a cylindrical tube, this implies that the variation in cell elongation following a change in radius and for a homogeneous material deformation should be $\Delta Q=\frac{1}{2}\ln [{R}^{+}/{R}^{-}]$, where *R*^−^ (*R*^+^) is the radius of the tube before (after) the deformation. We tested this relation by imposing a fast change in pressure on tubes of different radii, and by measuring the resulting change in tube radius and change in the measured average cell elongation Δ*Q*. For the measurement of Δ*Q* with a fast change in pressure, the junction angle distribution was obtained from bright-field images, instead of VE-cadherin stainings in the general case. Using images with both VE-cadherin staining and bright-field pictures, we find that the measured value of *Q* using VE-cadherin staining corresponds to approximately half its measured value using the bright-field image (Extended Data Fig. 6b(i)). Converting the value of *Q* computed from the bright-field image to its VE-cadherin-staining-based value, we finally found that the linear relation $\Delta Q=\frac{1}{2}\ln [{R}^{+}/{R}^{-}]$ was satisfied for a correction factor of *α*_*Q*_ = 0.8 (Extended Data Fig. 6b(ii)).

### Nuclei metrics and density measurements

Nuclei were segmented using the StarDist ImageJ plug-in^65^. Nuclei area and aspect ratio were extracted from each segmented object in ImageJ. Cell density was calculated by dividing the number of cells in a field of view by the area (accounting for channel curvature), for each position along the channel length. The mean cell area was calculated as the inverse of cell density.

### Proliferation assay

To assess the endothelial cell proliferation, EdU was added to the cell culture medium at a concentration of 10 μM. Cells were maintained in the EdU-containing culture medium for 8 h, either at 150 Pa or at 650 Pa, after which they were fixed and stained for DAPI and EdU-positive nuclei. The fraction of EdU-positive to EdU-negative nuclei provides a measure of the proliferation rate.

### Statistical analysis

The statistical unit corresponds to an experimental replicate, that is, a single microvessel. For Figs. 1–5 and Extended Data Figs. 1–6, all data are plotted as mean ± standard deviation, except for the probability distribution plots in which the line corresponds to the mean curve and the shadowed area corresponds to the standard error of the mean. All significance testing is based on an unpaired Student’s *t*-test, performed using MATLAB. *** denotes *P* < 0.001, ** denotes *P* < 0.01 and * denotes *P* < 0.05.

### Reporting summary

Further information on research design is available in the Nature Portfolio Reporting Summary linked to this article.

## Online content

Any methods, additional references, Nature Portfolio reporting summaries, source data, extended data, supplementary information, acknowledgements, peer review information; details of author contributions and competing interests; and statements of data and code availability are available at 10.1038/s41567-025-02847-3.

## Supplementary information

Supplementary Information Supplementary Sections 1 and 2.

Reporting Summary

Supplementary Video 1 Time-lapse images of the channel cross-section under a rapid pressure increase from 150 Pa to 650 Pa, showing an elastic expansion followed by a stable radius. Time, seconds. Scale bar, 50 µm.

Supplementary Video 2 Laser ablation experiment: fluorescence time-lapse images of LifeAct-endothelial cells on a soft collagen gel showing the endothelial actin network before and after longitudinal ablation, with a rapid opening of the wound, characteristic of high tissue tension in the circumferential direction. Time, seconds. Scale bar, 20 µm.

Supplementary Video 3 Monolayer stiffness measurement: bright-field time-lapse images of an endothelial tube on a soft gel subjected to a linear increase in pressure from 150 Pa to 1,000 Pa, undergoing circumferential expansion with a visible slow-down characteristic of a strain-stiffening behaviour. Time, seconds. Scale bar, 50 µm.

Supplementary Video 4 Three-dimensional reconstruction of the actin network of an endothelial tube at 150 Pa. Obtained from a *z* stack of fluorescence images of a monolayer stained with phalloidin, showing a longitudinal alignment of the actin stress fibres.

Supplementary Video 5 Three-dimensional reconstruction of the cells and nuclei of an endothelial tube at 150 Pa. Obtained from a *z* stack of fluorescence images of a monolayer stained with VE-cadherin (white) and DAPI (cyan), showing a longitudinal alignment of the cells and nuclei.

Supplementary Video 6 Three-dimensional reconstruction of the actin network of an endothelial tube at 650 Pa. Obtained from a *z* stack of fluorescence images of a monolayer stained with phalloidin, 7 h after pressure increase, showing a circumferential alignment of the actin stress fibres.

Supplementary Video 7 Three-dimensional reconstruction of the cells and nuclei of an endothelial tube at 650 Pa. Obtained from a *z* stack of fluorescence images of a monolayer stained with phalloidin, 7 h after pressure increase, showing a circumferential alignment of the cells and nuclei.

Supplementary Video 8 Variations in the actin nematic field along the channel length. Endothelial tubes stained with phalloidin, with the orientation of the actin stress fibres colour coded, at 0 h (top row), 7 h (second row), 24 h (third row) and 56 h (bottom row), showing the variability in fibre distribution along the channel length (each frame in the video is taken at a different *x* position).

## Source data

Source Data Fig. 1–5 and Source Data Extended Data Fig. 1–6 Statistical source data.

## Data Availability

Data for each plot are available in the Supplementary Information. Raw data are available from the corresponding authors upon reasonable request. Source data are provided with this paper.

## Code availability

Codes are available via GitLab at https://gitlab.unige.ch/salbreux-group/nicolas-cuny/endothelialtubemodel.
