# Supplementary material for: Interplay of actin nematodynamics and anisotropic tension controls endothelial mechanics
Source: Nat Phys. 2025 Apr 18;21(6):999–1008. doi: 10.1038/s41567-025-02847-3 (PMC12176649; doi:10.1038/s41567-025-02847-3)
Supplement: Supplementary file 1 — Supplementary Sections 1 and 2. [file 41567_2025_2847_MOESM1_ESM.pdf]

nature physics

Supplementary information https://doi.org/10.1038/s41567-025-02847-3

Interplay of actin nematodynamics and anisotropic tension controls endothelial mechanics

In the format provided by the authors and unedited

Contents

1 Mechanical model of the endothelial tube at long time scales **2**
1.1 Mechanical model of endothelial tube 2
1.1.1 Differential geometry of a cylindrical tube 2
1.1.2 Dynamics of the actin fibers mean orientation tensor 3
1.1.3 Force balance 4
1.1.4 Constitutive equation for the tension 4
1.1.5 Constitutive equations for cell area and cell elongation dynamics 5
1.1.6 Dimensionless system of equations for tube radius, nematic order, cell area and elongation 6
1.1.7 Initial conditions and dynamics of pressure application 6
1.2 Model comparison to experimental data 8
1.2.1 Fitting procedure 8
1.2.2 Discussion of best fit results 9
1.2.3 Discussion of the inferred parameters value 10
1.2.4 Effect of the magnitude of applied pressure 11
1.2.5 Pressure arrest experiment 11
1.3 Discussion of the model 11
1.3.1 Stability analysis of steady-state solutions 11
1.3.2 Viscoelastic response of actin stress fibers 12
1.3.3 Contribution of collagen gel to long time-scale response of endothelial tube 13
1.3.4 Elastic behaviour of residual tension 14
1.3.5 Effect of tube curvature on actin stress fibers orientation 15
1.3.6 Coupling of actin stress fiber orientation dynamics on total tension 15
1.3.7 Model of the tissue as an isotropic, viscoelastic material 15
1.3.8 Isotropic viscoelasticity with anisotropic active tension 16

2 Mechanical model of the endothelial tube instantaneous strain-stiffening **19**
2.1 Model of the endothelial tube 19
2.1.1 Gent model 19
2.1.2 Contribution of the surrounding collagen gel 19
2.1.3 Fitting procedure 20
2.2 Results 20
2.2.1 Substrate-dependent properties of endothelial tubes 20
2.2.2 Subcellular determinants of endothelium properties. 21

Part 1 Mechanical model of the endothelial tube at long time scales

In the following, we introduce a mechanical model of nematic active surface that we propose to describe endothelial tubes. This model relies on a continuum theory that describes the tissue on scales larger than the single cell. We focus on the average behavior of endothelial tubes, represented in the model as cylinders with homogeneous cell area, cell elongation, nematic order and mechanical properties. The model is introduced in section 1.1. The fitting procedure to experimental data, and parameter values, are discussed in section 1.2. We discuss model hypothesis in section 1.3.

1.1 Mechanical model of endothelial tube

1.1.1 Differential geometry of a cylindrical tube

As the thickness of the endothelial tissue is small with respect to the dimension of the tube ($\approx 4\mu m \ll 60\mu m$), we consider the endothelium vessel as an infinitely thin tube of radius R and length L (Fig.4Ai). We take $(\boldsymbol{u}_x, \boldsymbol{u}_y, \boldsymbol{u}_z)$ a cartesian basis of the space such that $\boldsymbol{u}_z$ is oriented along the axis of the cylinder. The surface of the tissue can be parametrized by two variables θ and z such that the position vector of each point of the surface reads:

$$\boldsymbol{X}(\theta, z) = R\left(\cos\theta\, \boldsymbol{u}_x + \sin\theta\, \boldsymbol{u}_y\right) + z\, \boldsymbol{u}_z, \tag{1.1}$$

with $\theta \in [0, 2\pi]$ and $z \in [0, L]$. Associated to those two variables, we can define a local tangential basis $(\boldsymbol{e}_\theta, \boldsymbol{e}_z)$ at each point of the surface with:

$$\boldsymbol{e}_\theta = \partial_\theta \boldsymbol{X} = R\left(-\sin\theta\, \boldsymbol{u}_x + \cos\theta\, \boldsymbol{u}_y\right), \tag{1.2}$$

$$\boldsymbol{e}_z = \partial_z \boldsymbol{X} = \boldsymbol{u}_z, \tag{1.3}$$

and the local normal vector is:

$$\boldsymbol{n} = \cos\theta\, \boldsymbol{u}_x + \sin\theta\, \boldsymbol{u}_y. \tag{1.4}$$

In this basis, the metric, inverse metric and curvature tensors have the following expressions:

$$g_{ij} = \boldsymbol{e}_i \cdot \boldsymbol{e}_j = \begin{pmatrix} R^2 & 0 \\ 0 & 1 \end{pmatrix}, \tag{1.5}$$

$$g^{ij} = (g_{ij})^{-1} = \begin{pmatrix} 1/R^2 & 0 \\ 0 & 1 \end{pmatrix}, \tag{1.6}$$

$$C_{ij} = -(\partial_i \boldsymbol{e}_j) \cdot \boldsymbol{n} = \begin{pmatrix} R & 0 \\ 0 & 0 \end{pmatrix}, \tag{1.7}$$

and the Christoffel coefficients Γ^k_{ij} vanish.

We consider that the vessel remains cylindrical, with its length L fixed and its radius R changing in time. Thus, even if locally the endothelial cells can have tangential displacement, the mean flow at the surface of the cylinder is purely normal to the surface of the tissue, and the velocity is simply $\boldsymbol{v} = v_n \boldsymbol{n}$. The normal velocity is given by $v_n = \mathrm{d}R/\mathrm{d}t$. The strain-rate tensor then reads:

$$v_{ij} = C_{ij} v_n = \begin{pmatrix} R\frac{\mathrm{d}R}{\mathrm{d}t} & 0 \\ 0 & 0 \end{pmatrix}, \quad v_i{}^j = \begin{pmatrix} \frac{1}{R}\frac{\mathrm{d}R}{\mathrm{d}t} & 0 \\ 0 & 0 \end{pmatrix} \tag{1.8}$$

1.1.2 Dynamics of the actin fibers mean orientation tensor

Endothelial cells exhibit a network of actin stress fibers (Fig. 2Ci). The orientation of each stress fiber can be described by an unitary vector n_i. As we aim to build a mesoscopic theory, we construct the mean orientation tensor $n_{ij} = \langle n_i n_j \rangle$, where the operator $\langle . \rangle$ indicates the average over all the actin fibers. The tensor n_{ij} describes the orientation of a set of fibers with a single mesoscopic quantity. This tensor is symmetric, with trace equal to 1. The tensor n_{ij} can be decomposed into an isotropic and a traceless part as follows:

$$n_{ij} = \frac{g_{ij}}{2} + q_{ij}, \tag{1.9}$$

where q_{ij}, the traceless part of n_{ij}, measures the anisotropy of the fibers orientation.

In the following we consider that off-diagonal coefficients of n_{ij} and q_{ij} vanish, as experimental measurements indicate that their values are small (Extended Data Fig. 4Ai,Bi). Because q_{ij} has zero off-diagonal coefficients and is a traceless tensor, it has only one independent component $q = q_\theta^\theta = -q_z^z$:

$$q_{ij} = \begin{pmatrix} R^2 q & 0 \\ 0 & -q \end{pmatrix}, \; q_i{}^j = \begin{pmatrix} q & 0 \\ 0 & -q \end{pmatrix}. \tag{1.10}$$

The value of q varies between $-1/2$ and $1/2$: for $q = -1/2$ all fibers are aligned with the tube axis, whereas for $q = 1/2$ all fibers are oriented circumferentially.

Passive deformation by tissue shear. Does the change of orientation of actin fibers occurs simply by following the tissue shear, or is it driven by additional effects? To determine the contribution of material flow to actin stress fibers reorientation, we computed the evolution of q as a function of a numerically applied uniaxial strain in the circumferential direction, for different initial distributions of orientation. To generate initial distributions, we extracted a set of vectors oriented along actin stress fibers from immunostaining images of the actin filaments (Fig. 4B, Methods). We then applied a given uniaxial strain along the circumferential direction by deforming each vector individually. The order parameter q was computed from the new distribution of angles of the strained vector field. We repeated this operation for strains ranging from 0 to 100%, for six different initial actin immunostaining images, to evaluate q as a function of R (Fig. 4B). We then compared this prediction to the experimentally measured order parameters q (mean values taken from Fig. 4Di) for different radii (mean values taken from Fig. 4Dii). This comparison shows that the increase of q due to deformation by the material flow explains less than 20% of actually observed actin stress fibers reorientation; indicating that other effects are driving actin stress fibers dynamics. For simplicity, we thus neglect the effect of tissue shear on actin reorientation in the model.

Actin fibers mechanosensitivity. Experimental observations suggest that actin stress fibers are in an ordered phase (Fig. 2C), and that their orientation is sensitive to the direction of tension (Fig. 2C, 3). We therefore choose the following equation for the dynamics of actin fibers nematic order:

$$D_t q_{ij} = -\gamma \left(\frac{1}{2} q_{kl} q^{kl} - q_0^2 \right) q_{ij} + \beta \tilde{t}^r_{ij}, \tag{1.11}$$

where $1/\gamma$ is the characteristic relaxation time of q_{ij}, q_0 is a preferred order magnitude, β is a mechanosensitive coupling term to $\tilde{t}^r_{ij} = t^r{}_{ij} - t^{r\,k}{}_k \, g_{ij}/2$, the traceless part of the residual tension $t^r{}_{ij}$, introduced below in Eq. 1.18. The operator D_t denotes the corotational derivative, with expression for a symmetric tensor A_{ij}:

$$D_t A_{ij} = \partial_t A_{ij} + v^k \nabla_k A_{ij} + \omega_{ik} A^k{}_j + \omega_{jk} A_i{}^k, \tag{1.12}$$

with $\omega_{ij} = 1/2(\nabla_i v_j - \nabla_j v_i)$. The first term in the right-hand side of Eq. 1.11 sets a preferred magnitude q_0 for actin stress fibers ordering. In the second term, actin stress fibers are assumed to respond to tension anisotropy in the tissue and to align with the direction of maximal residual tension t^r_{ij}. Here we exclude the contribution of tension within the actin stress fibers themselves to this feedback effect. We consider in section 1.3.6 the case where a coupling to the total tension of the tissue is replacing the coupling to t^r_{ij}.

1.1.3 Force balance

In the following we consider that the tissue is subjected to an internal tangential tension t_{ij}. Neglecting inertia as biological systems generally operate at low Reynolds number, the force balance in the normal direction reads

$$C_{ij}t^{ij} = \Delta P, \tag{1.13}$$

where $\Delta P = P - P_{\mathrm{gel}}$ is the difference of pressure across the endothelial monolayer, equal to the difference between the luminal pressure and the surrounding gel pressure. We consider the pressure in the gel P_{gel} to be fixed and equal to 0, and therefore ignore possible contributions from the gel elasticity. We relax this assumption in section 1.3.3, and find based on measurement of the viscoelastic response of collagen gel (Extended Data Fig. 1B) that its contribution to the overall response to pressure is indeed small (See section 1.3.3 of this SI).

Since the curvature tensor has only one non-zero component $C_{\theta\theta}$ (Eq. 1.7), one obtains simply the law of Laplace:

$$t^{\theta}_{\theta} = R\Delta P. \tag{1.14}$$

In the following we assume that the pressure difference between the fluid in the tube and the surrounding gel, ΔP, and all other physical quantities, are invariant along the longitudinal axis of the tube. In practice a flow has to be maintained in the tube for cell survival, which creates a gradient of pressure along the tube longitudinal direction of $\simeq$ 100Pa (Methods). In the following we use average values of pressures inside the tube for our analysis.

1.1.4 Constitutive equation for the tension

We discuss here the constitutive equation for the tissue tension tensor t_{ij}. In proposing constitutive equations for this tension tensor, we adopted the following rationale. Following previous studies [1, 2], we first reasoned that resistance to tissue expansion could generally arise from elastic resistance to cell area or to cell elongation. In addition, the strong reorientation of actin fibers observed experimentally suggested that anisotropic tension arising from the actomyosin network could play a role.

However, experimental data showed that both the average cell area and average magnitude of cell elongation drop 56 hours after the application of the increased pressure to the tube (Fig. 2Div, 5Aii). If cells had a fixed value of preferred area and elongation, this would imply that elastic stresses related to area and elongation should also decrease over time. However, the vessel radius increases continuously over the time course of the experiment, such that the tension in the circumferential direction t^{θ}_{θ} must also increase, according to Eq. 1.14. This indicates that the tension in the tissue is not dominated by cell area and cell elongation elasticity. In addition, we note that the average cell density varies by a factor 4 in different tissues, suggesting that cell area is not strongly regulated (Fig. 5Aiii). Therefore, we propose that the tension generated by actomyosin fibers is a key player in the tissue mechanics.

Tension generated by actomyosin fibers. We assume that the tension within the network of actin stress fibers lays along the mean orientation of fibers:

$$t^{a}_{ij} = \zeta n_{ij}, \tag{1.15}$$

where ζ is the magnitude of the resulting tension. The tension has both an isotropic part (proportional to $g_{ij}/2$) and an anisotropic part (proportional to q_{ij}) with equal magnitudes. The simplest description of actin generated tension would be to consider that every fiber is generating the same constant tension ζ_f. In this case $\zeta = \rho\zeta_f$ would be constant, if we consider the density of fibers ρ to be fixed. However, we find that this would not be compatible with the dynamics of tube expansion under pressure. Indeed, $t^{\theta}_{\theta} = \zeta(q + 1/2)$ would then plateau at long time, as q reaches a maximum value after 24 hours (Fig. 2Civ) and ζ is constant. As noted above, the law of Laplace implies that t^{θ}_{θ} increases steadily in time. This suggests that actin stress fibers should generate a circumferential tension increasing in time. We therefore assume that actin stress fibers respond elastically to material deformation. If an individual actin fiber behaves as a spring of stiffness K_a and orientation n_i and

deforms with the tissue, the tension generated by this fiber is $s_{ij} = K_a s\, n_i n_j$, with s the elongational stretch of the spring. The relative velocity of the two extremities of the fiber due to the material flow is ${v_i}^k n_k$, such that its elongation rate is $D_t s = v^{kl} n_k n_l$, with $D_t s = \partial_t s + v^k \nabla_k s$. Inspired by this result for a single spring-like actin fiber, we choose the following constitutive equation for the tension generated by actin stress fibers:

$$t^a_{ij} = (\zeta_0 + K_a s)\, n_{ij}, \tag{1.16}$$

$$D_t s = v^{kl} n_{kl}. \tag{1.17}$$

where s corresponds to an average level of fiber elongational stretch and ζ_0 is the amplitude of the active tension generated by actin fibers.

Additional tension generated by the tissue. Laser ablation experiments show that a few minutes after the application of the additional pressure, the tension is strongly anisotropic, being higher along the circumferential direction (Fig. 1Bii), which is in contradiction with predictions of Eq. 1.16. Indeed, just after application of the pressure increase, actin fibers are still mainly oriented along the tube axis, implying that t^a_{ij} has higher magnitude along the longitudinal than circumferential direction of the tube. We therefore propose that the tension in the tissue does not only depend on actin stress fibers, and can be decomposed into two contributions:

$$t_{ij} = t^a_{ij} + t^r_{ij}\ , \tag{1.18}$$

corresponding respectively to the tension generated by the cellular actomyosin network, and a residual tension acting within the other constituents of the tissue. Isotropic tension arising from cell area elasticity would not change the tension anisotropy. Cell elongation elasticity will also not lead to circumferentially oriented tension just after pressure step application, because cell elongation is still mainly along the tube direction. Therefore, we assume that other elements in the tissue mechanically resist its deformation. These elements could for instance correspond to microtubules, cortical actin, cell membrane or intermediate filaments. Since a full description of these cytoskeletal elements at all time scales is out of reach, we describe its rheology by a simple Maxwell model, which retains the longest time scale of relaxation of the system from elastic to fluid behaviour. Therefore, we write the residual tension tensor as:

$$(1 + \tau D_t)\, t^r_{ij} = \mu v_{ij}, \tag{1.19}$$

where τ is the characteristic time of the Maxwell model, μ a viscosity and D_t the corotational derivative defined in Eq. 1.12. We discuss this choice of constitutive relation for t^r_{ij} in Sec. 1.3.4.

1.1.5 Constitutive equations for cell area and cell elongation dynamics

Here we discuss the dynamics of the mean cell area a and mean cell elongation Q_{ij} which are coupled to the tissue expansion.

Area dynamics. The area dilatation rate of an infinitesimal piece of a surface is equal to the local isotropic growth rate v^k_k of this surface. Cells also divide at a rate k_d, that we assume to be constant. Assuming that cell apoptosis or delamination can be neglected, the dynamics of the mean cell area then reads [1, 2]:

$$\frac{\partial_t a}{a} = v^k_k - k_d. \tag{1.20}$$

Cell elongation dynamics. We now discuss the dynamics of the cell elongation tensor Q_{ij}. The surface deformation contributes to cell elongation through its deviatoric part $\tilde{v}_{ij} = v_{ij} - v^k_k g_{ij}/2$. However, this purely geometric contribution is not sufficient to explain the cell elongation measured 7 and 24 hours after additional pressure increase (Fig. 5Biii). Indeed we observe at these two times an excess of circumferential cell elongation measured experimentally relative to the value predicted by the geometrical contribution only. Moreover, we observe a strong correlation between cell elongation and actin orientation (Fig. 5Bii), suggesting that the cell elongation tensor Q_{ij} follows the dynamics of the actin stress fiber nematic tensor q_{ij}. We therefore propose the following dynamical equation for Q_{ij}:

$$D_t Q_{ij} = \tilde{v}_{ij} - \lambda(Q_{ij} - \alpha q_{ij}) \tag{1.21}$$

where $1/\lambda$ is the characteristic time of alignment of cell elongation with the actin stress fibers, and α is a proportionality factor between the cell elongation and actin stress fibers nematic tensor. The first term in the right-hand side corresponds to cell elongation changes arising from the tissue anisotropic shear, and the last term in the right-hand side arises from cellular rearrangements in the tissue tending to relax the cell elongation tensor towards the actin fiber tensor. [1, 2].

1.1.6 Dimensionless system of equations for tube radius, nematic order, cell area and elongation

To obtain dimensionless equations, we choose R_0, the radius of the tube at $t = 0$, before the application of the additional pressure, as length unit, τ, the Maxwell viscoelastic time, as time unit, and ζ_0, the constant actin tension, as unit of tension (Table 1.1).

Physical quantity	Unit
Length	R_0
Time	τ
Tension	ζ_0

Table 1.1: Physical variables used for non-dimensionalisation.

We transform variables and parameters such as $R/R_0 \to R$, $t/\tau \to t$, $R_0\Delta P/\zeta_0 \to \Delta P$, $K_a/\zeta_0 \to K_a$, $\mu/(\tau\zeta_0) \to \mu$, $\gamma\tau \to \gamma$, $\tau\beta\zeta_0 \to \beta$, $\tau k_d \to k_d$, $\tau\lambda \to \lambda$. For convenience we also use $a/a(0^-) \to a$. We also introduce $\sigma = t^{r\,\theta}{}_{\theta}/\zeta_0$ and $Q = Q_\theta^\theta = -Q_z^z$, as Q_{ij} is a traceless tensor. We consider that off-diagonal coefficients of Q_{ij} vanish, as experimental measurements indicate that their value is small (Extended Data Fig. 4Aii, Bii). We then obtain the following system of dimensionless equations:

$$R\Delta P = \sigma + (1 + K_a s)\left(q + \frac{1}{2}\right), \tag{1.22}$$

$$\left(1 + \frac{\mathrm{d}}{\mathrm{d}t}\right)\sigma = \frac{\mu}{R}\frac{\mathrm{d}R}{\mathrm{d}t}, \tag{1.23}$$

$$\frac{\mathrm{d}s}{\mathrm{d}t} = \left(q + \frac{1}{2}\right)\frac{1}{R}\frac{\mathrm{d}R}{\mathrm{d}t}, \tag{1.24}$$

$$\frac{\mathrm{d}q}{\mathrm{d}t} = -\gamma\left(q^2 - q_0^2\right)q + \frac{1}{2}\beta\sigma, \tag{1.25}$$

$$\frac{1}{a}\frac{\mathrm{d}a}{\mathrm{d}t} = \frac{1}{R}\frac{\mathrm{d}R}{\mathrm{d}t} - k_d, \tag{1.26}$$

$$\frac{\mathrm{d}Q}{\mathrm{d}t} = \frac{1}{2R}\frac{\mathrm{d}R}{\mathrm{d}t} - \lambda(Q - \alpha q). \tag{1.27}$$

The dimensionless expressions of physical quantities and coefficients are given in Table 1.2.

1.1.7 Initial conditions and dynamics of pressure application

We discuss here initial conditions and the dynamics of pressure ΔP which have to be specified to solve Eqs. 1.22-1.27. In the experimental protocol, cells are seeded on the tube surface and allowed to spread on the gel and proliferate until reaching confluence. The time of confluence is defined as $t = t_c = -24$hours. After confluence, the cells form a low-permeability barrier and a small pressure difference of $\Delta P_0 \simeq 150$Pa is then established within the tube. This low pressure stems from the minimal fluid flow in the tube necessary to maintain cells alive. This state is maintained during 24 hours to mature the monolayer. The tube radius increases slightly by $\simeq 5\%$ in 24 hours at this low pressure. At $t = 0$, a larger pressure difference ΔP_m is applied.

We consider that just before reaching confluence ($t = t_c^-$ with $t_c = -24$h), the assembly of disjointed cells is not impermeable to the luminal fluid and the pressure difference ΔP vanishes. There is then no circumferential tension in the tissue, and we further assume that actin fibers are unstretched, $s = \sigma = 0$. The first term in

			Dimensionless expression
Physical variables	R	R/R_0
	t	t/τ
	q	q
	ΔP	$R_0\Delta P/\zeta_0$
	σ	σ/ζ_0
	a	$a/a(0^-)$
Parameters of the model	τ	1
	ζ_0	1
	K_a	K_a/ζ_0
	μ	$\mu/(\tau\zeta_0)$
	γ	$\tau\gamma$
	q_0	q_0
	β	$\tau\beta\zeta_0$
	k_d	τk_d
	λ	$\tau\lambda$
	α	α

Table 1.2: List of physical variables and model parameters, and corresponding dimensionless expression.

the right-hand side of Eq. 1.25 allows both $q = \pm q_0$ as a reference state. Here we consider that actin fibers in the tube initially prefer to align along the tube axis, possibly due to minimization of bending energy, thus selecting $q = -q_0$ (See section 1.3.5 of this SI). We also assume that before confluence cells are at their preferred elongation, i.e $Q(t_c^-) = -\alpha q_0$. For simplicity, we consider that the pressure difference ΔP increases from zero to ΔP_0 at the establishment of confluence ($t = t_c$) on a time scale much faster than the characteristic times of the model, such that only R, s and the elastic part of σ respond to the pressure change and that a and Q jump according to the tissue deformation. Then just after confluence ($t = t_c^+$), one obtains:

$$s(t_c^+) = \left(-q_0 + \frac{1}{2}\right) \ln \frac{R(t_c^+)}{R(t_c^-)}, \tag{1.28}$$

$$\sigma(t_c^+) = \mu \ln \frac{R(t_c^+)}{R(t_c^-)}, \tag{1.29}$$

$$R(t_c^+)\Delta P_0 = \sigma(t_c^+) + (1 + K_a s(t_c^+)) \left(-q_0 + \frac{1}{2}\right), \tag{1.30}$$

$$a(t_c^+) = \frac{a(t_c^-)R(t_c^+)}{R(t_c^-)}, \tag{1.31}$$

$$Q(t_c^+) = Q(t_c^-) + \frac{1}{2} \ln \frac{R(t_c^+)}{R(t_c^-)} \tag{1.32}$$

$R(t_c^+)$ is then determined by replacing $s(t_c^+)$ and $\sigma(t_c^+)$ by their expression as a function of $R(t_c^+)$ (Eqs. 1.28 and 1.29) in Eq. 1.30 and numerically solving the resulting equality. The value of all other quantities at $t = t_c^+$ are determined by $R(t_c^+)$. We then solve the dynamical equations Eqs. 1.22-1.27 with luminal pressure $\Delta P = \Delta P_0$, for $t_c^+ \leq t \leq 0^-$. Value of the variables R, q, σ and s at $t = 0^-$ are obtained from this solution. The normalised value of R at $t = 0^-$ is imposed to be equal to 1 by rescaling the solution. We consider that the pressure increases at $t = 0$ from ΔP_0 to ΔP_m much faster than the characteristic times of the model such that only R, s and the elastic part of σ change value to adapt to the change of pressure, and a and Q jump according to the tissue deformation. This gives:

$$s(0^+) = s(0^-) + \left(q(0^-) + \frac{1}{2}\right) \ln \frac{R(0^+)}{R(0^-)}, \tag{1.33}$$

$$\sigma(0^+) = \sigma(0^-) + \mu \ln \frac{R(0^+)}{R(0^-)}, \tag{1.34}$$

$$R(0^+)\Delta P_m = \sigma(0^+) + (1 + K_a s(0^+))\left(q(0^-) + \frac{1}{2}\right), \tag{1.35}$$

$$a(0^+) = a(0^-)\frac{R(0^+)}{R(0^-)}, \tag{1.36}$$

$$Q(0^+) = Q(0^-) + \frac{1}{2}\ln\frac{R(0^+)}{R(0^-)} \tag{1.37}$$

and q is continuous, $q(0^+) = q(0^-)$. As done previously, Eq. 1.35 can be used to determine the value of $R(0^+)$ that sets the value of all other quantities at $t = 0^+$. We then numerically solve the dynamical equations 1.22-1.27 for $t \geq 0$.

1.2 Model comparison to experimental data

1.2.1 Fitting procedure

Fitting of tube radius and actin order parameter dynamics. We performed a fitting procedure to infer the parameters of the model that reproduce the measured behaviour of the dimensionless values of R and q (in practice we simulate the dynamics of the system taking $R(t_c^-) = 1$ and renormalize *a posteriori* the radius by its value at $t = 0^-$). We use a Nelder-Mead algorithm to minimize the following cost function:

$$C(\{p\}) = A \times \left[R(\{p\}, t_c^-) - R_c\right]^2 + \sum_{i=1}^{N_R}\left(\frac{R(\{p\}, t_i/\tau) - \bar{R}_i/R_0}{\bar{R}_i/R_0}\right)^2 + \sum_{\alpha=1}^{N_q}\left(\frac{q(\{p\}, t_\alpha/\tau) - \bar{q}_\alpha}{q^*}\right)^2, \tag{1.38}$$

where $\{p\}$ is a set of value of the parameters of the model and $R(\{p\}, t)$ (resp. $q(\{p\}, t)$) the value of the variable R (resp. q) at time t when the model is solved numerically with parameters value $\{p\}$.

Experimental data consist in two set of measurements: $\bar{R}_i$ the value of the radius measured at time t_i, and $\bar{q}_\alpha$ the value of q measured at time t_α. There are N_R measured values of the radius R and N_q measured values of the order parameter q. q^* is the mean value of measurements of q performed at $t = 0^-$ and R_c the experimental value of the normalized tube radius when tissue reaches confluence. To enforce a $\simeq 5\%$ radius increase between t_c^- and $t = 0^-$ as observed in experiments we take $R_c = 0.96$ and $A = 2000$.

In order to assess the sensitivity of our model to its different parameters, we use a bootstrap method [3]. For each radius and actin orientation measurement, we define the deviations $\varepsilon_{R,i} = (\bar{R}_i - R_i^*)/R_0$ and $\varepsilon_{q,i} = \bar{q}_i - q_i^*$, with R_i^* (resp. q_i^*) the value of the average curve of experimental measurements of R (resp. q) at time t_i. We then generated a virtual set of experimental data $\{\bar{R}_i' = R_i^* + \delta_{R,i} \,|\, i = 1...N_R\}$ and $\{\bar{q}_i' = q_i^* + \delta_{q,i} \,|\, i = 1...N_q\}$ with $\{\delta_{R,i}\}$ (resp. $\{\delta_{q,i}\}$) a set of random numbers picked in $\{\varepsilon_{R,i}\}$ (resp. $\{\varepsilon_{q,i}\}$). The cost function (1.38) is minimized with a Nelder-Mead algorithm, replacing $\bar{R}_i$ and $\bar{q}_\alpha$ by $\bar{R}_i'$ and $\bar{q}_\alpha'$ within Eq. 1.38, and using the parameters of the original fit as initial guess for the Nelder-Mead algorithm of the new fit. This procedure was reiterated 2000 times to obtain a distribution of fit parameters. The mean value and standard deviation of the distribution of each parameter is reported in Table 1.3. Parameters generally have low ($\sim 10\%$) uncertainty, except for the inverse timescale of response of nematic order, γ, which is less precisely determined (Extended Data Fig. 4C).

Fitting of cell area dynamics. The optimal value of the cell division rate k_d is determined as follows, from experimental measurements of the cell mean area a. Integrating Eq. 1.26 yields the following relation between mean cell area and vessel radius:

$$a(t) = R(t)e^{-k_d t}. \tag{1.39}$$

The value of k_d was adjusted to fit experimental values of $\bar{a}_i/a_0$ by $R(\{p\}, t_i/\tau)e^{-k_d t_i/\tau}$. Here $\bar{a}_i$ is the value of mean cell area measured at time $t_i = 0, 7, 24$ and 56h, a_0 the mean value of measured mean cell areas at $t = 0^-$, $R(\{p\}, t_i/\tau)$ the value of the normalized radius predicted by the model, for optimal values of parameters p and τ, as determined by the previous fit on R and q. The *curve_fit* function of Scipy python package was used to determine the optimal value of k_d and its uncertainty, reported in Table 1.3. The corresponding best fit for the evolution of the cell area as a function of time is shown in Fig. 5Aii.

Measured parameters	$R_0 = 64 \pm 3\,\mu m$
	$\Delta P_m = 650 \pm 50\,Pa$
Dimensionless parameters	$\Delta\hat{P}_m = 1.4\ (1.4 \pm 0.1)$
	$\hat{\mu} = 9.9\ (10.5 \pm 1.1)$
	$\hat{K}_a = 7.6\ (7.3 \pm 1.4)$
	$\hat{\gamma} = 2.9\ (4.3 \pm 5.1)$
	$q_0 = 0.26\ (0.26 \pm 0.02)$
	$\hat{\beta} = 0.38\ (0.38 \pm 0.08)$
	$\hat{k}_d = 0.037 \pm 0.091$
	$\hat{\lambda} = 4.3\ (22.9 \pm 46.9)$
	$\alpha = 0.47\ (0.48 \pm 0.07)$
Dimensional parameters	$\tau = 3.4h\ (3.4 \pm 0.6h)$
	$\zeta_0 = 0.03N/m\ (0.030 \pm 0.003N/m)$
	$K_a = 0.22N/m\ (0.22 \pm 0.03N/m)$
	$\mu = 0.98N/m.h\ (1.1 \pm 0.1N/m.h)$
	$K = \mu/\tau = 0.29N/m\ (0.31 \pm 0.03N/m)$
	$\gamma = 0.87h^{-1}\ (1.3 \pm 1.3h^{-1})$
	$\beta = 3.8N^{-1}.m.h^{-1}\ (3.7 \pm 0.5N^{-1}.m.h^{-1})$
	$k_d = 0.011 \pm 0.003h^{-1}$
	$\lambda = 1.3h^{-1}\ (6.7 \pm 14.0h^{-1})$
	$1/\lambda = 0.8h\ (1 \pm 1.1h)$

Table 1.3: Table of fitted parameters. Hatted symbols indicated dimensionless parameters according to Table 1.2 while unhatted symbols indicate parameters with physical dimensions. Inside parenthesis: mean value ± the standard deviation of parameter distributions obtained by bootstrap method (except for k_d).

Fitting of cell elongation dynamics. Following a similar procedure, best fit values are determined for the parameters λ and α involved in the dynamics of cell elongation. The cost function used for the fit now reads:

$$C_Q(\lambda, \alpha) = \sum_{i=1}^{N_Q} \left(Q\left(\lambda, \alpha, \frac{t_i}{\tau}\right) - \bar{Q}_i \right)^2, \tag{1.40}$$

with $Q(\lambda, \alpha, t)$ the value of Q at time t computed from Eq. 1.27 for the specified values of λ and α with initial condition $Q(0^-) = Q_0$, where Q_0 is the average of experimental values of Q at $t = 0^-$. The experimental values of cell elongation are $\{\bar{Q}_i\}_{i=1..N_Q}$, with t_i the time at which $\bar{Q}_i$ has been measured and N_Q the number of measurements. All parameters of the mechanical model (Eqs. 1.22-1.25) have been fixed to their optimal value determined by the mechanical model fit. The dynamics of Q with optimal values of λ and α is shown in Fig. 5Biii.

We have performed a sensitivity analysis of the parameters λ and α, as described in the paragraph above "Fitting of tube radius and actin order parameter dynamics". The set of deviations of experimental data from their average values is used to generate new virtual data, for which we repeat the fit procedure with the cost function (1.40). Parameter distributions are obtained by reiterating this procedure 2000 times. Mean value and standard deviation of parameter distributions are reported in Table 1.3. While α is strongly constrained (corresponding to a well-defined ratio between cell elongation and nematic order), λ has a large uncertainty, as sufficiently large values ensure that the cell elongation is slaved to nematic order, a limit which accounts well for experimental data (Extended Data Fig. 4D).

1.2.2 Discussion of best fit results

Optimal values of model parameters are given in Table 1.3.

Dynamics of tube radius and actin order parameter. The dynamic evolution of R and q is well captured using these best fit parameters (Fig. 4Di,ii). At short times after the pressure step, the residual tension t^r_{ij}

resists deformation elastically, which leads to circumferential reorientation of actin fibers (Fig. 4C, Div). This contribution then relaxes on a characteristic time τ due to the viscoelastic behaviour. The actin generated tension t^a_{ij}, now oriented circumferentially, then resists tissue expansion, such that at long time the dynamics of the tube is dominated by actin contribution (Fig. 4C, Div). To illustrate this point, we show the dynamics of the system when the elastic contribution of tension in actin fibers is removed, $K_a = 0$ (with all other parameters kept at the same value, Fig. 4Di,ii). In this case, the radius diverges in a few hours. We also highlight the importance for actin fibers to circumferentially reorient by simulating the dynamics of the system with actin-tension coupling term $\beta = 0$ (with all other parameters kept at the same value, Fig. 4Di,ii). In this case the radius also increases quickly, showing the importance of fibers aligning with the flow to resist the expansion of the tube.

Dynamics of tissue tension. The evolution of the components t^θ_θ and t^z_z in the model is shown in Fig. 4Diii. In the model, the longitudinal tension t^z_z is initially larger than the circumferential tension t^θ_θ. The difference is larger than detected by laser ablation (Fig. 1Bii, Extended Data Fig. 1A), possibly due to the weak longitudinal anisotropy of the high-density monolayers used for these experiments. At $t = 0^+$, t^θ_θ jumps by a larger amount than t^z_z such that the tension just after pressure step application is strongly anisotropic in circumferential direction, in agreement with laser ablation experiments.

Dynamics of cell elongation. The coupling between cell elongation and actin fiber elongation is resulting in an excess of elongation at 7 and 24 hours compared to the deformation expected from tissue shear, as observed experimentally (Fig. 5Biii). We also show the dynamics of Q for a pressure of 150Pa, starting at $t = t_c$ with the initial condition $Q(t_c^-) = -\alpha q_0$. The model however does not account for a drop of elongation at 56 hours. We suspect that this drop could be due to a coupling of cell elongation magnitude to the cell area, possibly via the oriented divisions reported in experiments, as cells with small areas are observed to be preferentially isotropic.

1.2.3 Discussion of the inferred parameters value

The characteristic active tension generated by actin is $\zeta_0 \approx 0.03 \pm 0.003 N/m$. The characteristic tension generated by its resistive part is $\sim K_a s_{max} \approx 0.06 N/m$, with $s_{max} \approx 0.25$, the maximum value of s (Extended Data Fig. 4F). The characteristic tension generated by the rest of the tissue is $\sim (\mu/\tau)\ln(R_0^+) \approx 0.04 N/m$, with R_0^+ the normalized radius of the vessel just after the increase of pressure to 650Pa. Each of those three contributions are of the same order of magnitude, towards large values of the previously reported range of tensions [4, 5]. This is broadly consistent with experimental observations that the stiffness of endothelial or epithelial tissues is divided by approximately two when actin is depolymerized [6, 7].

The characteristic time of actin reorganisation $\sim 1/\gamma \approx$ 70min is of the same order of magnitude as previously reported values of 5-40min of actin or cellular tension reorientation under cycling stretching [8, 9, 10, 11]. The non-dimensional coefficient $2q_0^2\gamma\tau/(\beta\mu) \approx 0.11$ is a characteristic strain inducing actin reorientation away from its original orientation. This is comparable to observations that a 10% strain is enough to induce actin fibers reorientation in endothelial cells [12]. We note however that the bootstrap analysis indicates that γ is determined with a large uncertainty. Imposing $\gamma = 0$ in the fitting procedure does not account well for experimental data, but large values of γ are still acceptable (Extended Data Fig. 4C).

The characteristic time of cell elongation relaxation towards actin orientation is $\sim 1/\lambda \approx$ 45min ($1/\lambda =$ 1 ± 1.1h, mean and standard deviation of bootstrap analysis). This small time scale relative to the total time of the experiment (56 hours) implies that Q correlates well with q, in accordance to the observed correlation throughout the experiment (Fig. 5Bii). Larger values of λ, corresponding to faster relaxation time $1/\lambda$ compared to 45min-1h, also account for experimental data, as in that case the dynamics of the elongation Q follows closely the dynamics of the nematic order q (Extended Data Fig. 4D). This results in a large uncertainty on the evaluation of λ.

The doubling time of cell is $\sim \ln(2)/k_d \approx 60h$, in agreement with experimental measurements of the number of divisions between 0 and 7 hours (Methods), which are consistent with reported values for comparable cell densities [13, 14].

1.2.4 Effect of the magnitude of applied pressure

To investigate the effect of the applied pressure at $t > 0$ on the tube expansion dynamics, we solve the model numerically for pressures of $\Delta P_m = 450$ Pa and 850 Pa, in addition to the value of $\Delta P_m = 650$ Pa used for fitting experimental data. The first stage of imposed pressure difference $\Delta P = 150$ Pa for $t_c < t < 0$ remains unchanged, such that for all pressures the tube is considered to be in the same state at $t = 0^-$. We show in Extended Data Fig. 4H the dynamics of the system for different pressures. Comparison of these results with experimental measurements of R, q, a and Q for different pressures, 7 hours after the pressure step (Fig. 5C), show that the model is in good agreement with experimental values.

1.2.5 Pressure arrest experiment

Here we investigate the change in tube radius following experiments where the applied pressure ΔP is reduced abruptly after 7 hours of tube expansion, from the reference value ΔP_m to the residual pressure ΔP_0 (Fig. 2Bi). We assume that the reduction in pressure occurs on a time scale much smaller than characteristic times of the model. Then only R, s and the elastic contribution of σ can immediately respond to the change of pressure. The state of the system just after the pressure arrest is then obtained by solving the following relations:

$$s^+ = s^- + (q^- + \frac{1}{2}) \ln \frac{R^+}{R^-}, \tag{1.41}$$

$$\sigma^+ = \sigma^- + \mu \ln \frac{R^+}{R^-}, \tag{1.42}$$

$$R^+ \Delta P_0 = \sigma^+ + (1 + K_a s^+)(q^- + \frac{1}{2}), \tag{1.43}$$

where the upperscript − (resp. +) accounts for the value of the variables just before (resp. after) the pressure arrest.

We find that the pressure release leads to a relaxation of the tube radius of similar magnitudes between theory and experiment. Experimentally, the instantaneous relative change of radius following pressure variation is smaller at 7 hours than at 0 hour. In the model however we find that this change is predicted to be rather constant (Extended Data Fig. 4E). This discrepancy may be linked to strain-stiffening properties of the tissue which we have not considered here for simplicity.

1.3 Discussion of the model

In this section we discuss the stability of steady-state cylindrical solutions of the nematic mechanical model introduced in section 1.2. We then discuss the different model hypothesis by considering alternative constitutive equations for the actin stress fibers nematodynamics and tissue tension, summarized in Table 1.4. This analysis allows to identify a minimal set of essential elements to account for experimental observations.

1.3.1 Stability analysis of steady-state solutions

Here we discuss steady-state solutions of the model for cylindrical tubes. At steady state, the tension t^r_{ij} vanishes, and the order parameter $q = \pm q_0$ (we consider here solutions where the alignment is circumferential or longitudinal). In this subsection, we use dimensional equations and assume $\beta \geq 0$, $\gamma > 0$, $\Delta P > 0$. Mechanical balance imposes that:

$$R_e \Delta P = t^\theta_\theta = (\zeta_0 + K_a s_e) \left(\frac{1}{2} \pm q_0 \right) \tag{1.44}$$

with R_e and s_e the tube radius and fiber strain at steady-state.

To test for the stability of this solution, we consider a perturbation $R = R_s + \delta R$, $s = s_e + \delta s$, $q = q_e + \delta q$ with $q_e = \pm q_0$. At linear order in the perturbation, one obtains

$$(1 + \tau \partial_t) \partial_t \delta q = -2\gamma q_0^2 (1 + \tau \partial_t) \delta q + \frac{\beta \mu}{2} \frac{\partial_t \delta R}{R_e} \tag{1.45}$$

$$(1+\tau\partial_t)\frac{\delta R}{R_e}\left(\frac{R_e\Delta P}{K_a}-\left(\frac{1}{2}+q_e\right)^2\right)=\frac{\mu}{K_a}\frac{\partial_t\delta R}{R_e}+\frac{\zeta_0+K_a s_e}{K_a}(1+\tau\partial_t)\delta q\ . \tag{1.46}$$

Performing a Laplace transform, we can ask when perturbations relax exponentially, corresponding to a stable solution. We find that positive pressure values satisfying

$$\Delta P < \frac{K_a\left(\frac{1}{2}+q_e\right)^2}{R_e} \tag{1.47}$$

give rise to stable solutions. This also corresponds to a range of stable strain values, at positive pressure:

$$-\frac{\zeta_0}{K_a} < s_e < \frac{1}{2}+q_e-\frac{\zeta_0}{K_a}. \tag{1.48}$$

The solution where fibers are circumferential ($q_e = q_0$) is therefore more stable than the solution where fibers are longitudinal ($q_e = -q_0$). With parameters in Table 1.3, this corresponds to a maximal strain $s_e \simeq 0.62$ for circumferential fibers alignment and $s_e \simeq 0.1$ for longitudinal alignment. This highlights the role of actin fibers reorientation in our model of vessel mechanics. Indeed, with longitudinal alignment stable solutions only exist for low strain until reaching a relatively low value s_e. As a result, when setting the tension coefficient β coupling actin orientation to tissue tension to zero, the tube radius quickly diverges (Fig. 4Dii) instead of simply reaching a higher steady value. However, even with circumferentially-oriented actin stress fibers, stable solutions disappear for large enough strain, indicating that the tube will not reach an equilibrium radius if the pressure crosses a threshold value. We find numerically that for pressures greater than $\simeq$ 780Pa the strain of actin fibers overcomes its critical value and the tube radius does not reach steady value (Extended Data Fig. 4Hi).

1.3.2 Viscoelastic response of actin stress fibers

In the constitutive equation for actin stress fibers (Eq. 1.16) we assumed that actin fibers respond elastically to deformation of the tube. This implies that actin fibers have a infinite memory of their initial reference state. Cellular processes may however lead to a loss of this memory. For instance, the actin cytoskeleton undergoes dramatic remodeling during cell division, with the disassembly of stress fibers during mitosis (Extended Data Fig. 5B). A simple way to take into account such memory loss is to consider a viscoelastic response of actin stress fibers:

$$t^a_{ij} = (\zeta_0+\sigma_a)n_{ij}, \tag{1.49}$$

$$(1+\tau_a D_t)\sigma_a = \mu_a v^{kl}n_{kl}, \tag{1.50}$$

with $\mu_a = \tau_a K_a$, where τ_a is actin tension relaxation time. We solve the dynamics equations as described in sections 1.1.6 and 1.1.7, assuming that $\sigma_a(t_c^-) = 0$.

We fitted experimental data of tube radius and actin orientation by optimizing parameters of this model through a Nelder-Mead algorithm minimizing the following cost function:

$$C(\{p\}) = A\times\left[R(\{p\},t_c^-)-R_c\right]^2+\frac{1}{N_{bin}}\sum_{i=1}^{N_{bin}}\left(\frac{R(\{p\},t_i)-R_{bin}(t_i)}{R_{bin}(t_i)}\right)^2+\frac{1}{N_q}\sum_{\alpha=1}^{N_q}\left(\frac{q(\{p\},t_\alpha/\tau)-\bar{q}_\alpha}{q^*}\right)^2, \tag{1.51}$$

where $\{p\}$ is a set of value of the constitutive equations parameters and $R(\{p\},t)$ the value of the radius at time t when its dynamics is solved numerically with parameters value $\{p\}$. $R_{bin}(t_i)$ is the average value of experimental radius value at time $t \in [t_i-3h, t_i+3h]$, except for $t_i = 0$ where $R_{bin}(0)$ corresponds to the average value of experimental radius values just after the pressure increase. We took $N_{bin} = 9$ and $t_i = 0, 3, 7, 10, 24, 28, 32, 47, 56$ hours.

We find that the model still accounts fairly well for experimental data, and that the tube radius does not reach a steady-state anymore (Extended Data Fig. 5A). The fitted value $\tau_a \approx$ 100h is close to the inverse division rate $1/k_d \approx$70h, the characteristic time at which cell division should relax the strain memory of actin fibers. This suggests that taking actin stress fibers as behaving elastically is a reasonable simplification on a time scale of 56 hours.

1.3.3 Contribution of collagen gel to long time-scale response of endothelial tube

To test whether the surrounding gel could have a significant contribution to the dynamics of the system, we consider the tissue model described in Sec. 1.1, now taking into account the mechanical contribution of the gel. The normal force balance then takes the form:

$$t_\theta^\theta = R(\Delta P + \sigma_{rr}(R)), \tag{1.52}$$

where $\sigma_{rr}(R)$ is the radial component of the stress tensor of the gel in cylindrical coordinates, taken at $r = R$ at the contact point with the tube. Based on rheological characterization of the gel (Extended Data Fig. 1B), we describe the gel as a Maxwell-like viscoelastic material. This description is a simplified, approximate description of the actual rheology of the gel, which is more complex on short time scale (Extended Data Fig. 1B). We also neglect the residual stress exhibited by stress-relaxation curves as this stress is at most equal to 40Pa (Extended Data Fig. 1Bi) which is small compared to the applied pressure in our experiments (650Pa). With this simplification, the stress inside the gel is described by the following constitutive equation, with greek indices corresponding to three-dimensional cartesian coordinates:

$$\left(1 + \tau_g D_t^g\right) \sigma_{\alpha\beta} = \mu_g \left(v_{\alpha\beta}^g + \frac{\nu_g}{1 - 2\nu_g} v_{kk}^g \delta_{\alpha\beta} \right), \tag{1.53}$$

with ν_g the Poisson ratio of the gel, τ_g its characteristic time, $\mu_g = \tau_g E_g/(1 + \nu_g)$ its viscosity, E_g its Young modulus and $v_{\alpha\beta}^g$ the strain-rate tensor inside the gel. The corotational derivative in the gel reads:

$$D_t^g A_{\alpha\beta} = \partial_t A_{\alpha\beta} + v_\gamma^g \partial_\gamma A_{\alpha\beta} + \omega_{\alpha\gamma}^g A_{\gamma\beta} + \omega_{\beta\gamma}^g A_{\alpha\gamma}, \tag{1.54}$$

with v_α^g the velocity field inside the gel and $\omega_{\alpha\beta}^g = (\partial_\alpha v_\beta^g - \partial_\beta v_\alpha^g)/2$ the vorticity, which vanishes in the geometry considered here. Fitting the relaxation curves of stress in Extended Data Fig. 1Bi by exponentials, we were able to estimate τ_g for different prestrains (Extended Data Fig. 5Ciii). Considering that the gel deforms only radially, the non-zero components of its strain-rate tensor reads, using cylindrical coordinates,

$$v_{rr}^g(r, \theta) = \partial_r v_r^g, \tag{1.55}$$

$$v_{\theta\theta}^g(r, \theta) = \frac{v_r^g}{r}, \tag{1.56}$$

where r and θ are the cylindrical coordinates. In addition, the radial force balance in the gel reads

$$\partial_r \sigma_{rr} = \frac{1}{r} \left(\sigma_{\theta\theta} - \sigma_{rr}\right). \tag{1.57}$$

We first discuss the contribution of the gel to the response to pressure steps, when the tissue reaches confluence ($t = t_c$) and when the pressure inside the tube is increased ($t = 0$). We consider that during these steps, the gel responds elastically. As the characteristic time of the gel is of the same order of the establishment time of the step of pressure at time $t = 0$ ($\sim 90s$), elastic stresses may actually have time to relax; further reinforcing our conclusion that stresses in the gel do not affect the endothelial tube deformation. The constitutive equation for the stress tensor in the gel then becomes

$$\partial_t \sigma_{\alpha\beta} = \frac{E_g}{1 + \nu_g} \left(v_{\alpha\beta}^g + \frac{\nu_g}{1 - 2\nu_g} v_{\gamma\gamma}^g \delta_{\alpha\beta} \right), \tag{1.58}$$

where we neglect the contribution of the advection term, as the deformation of the gel is small (less than 10%). Using Eqs. 1.58, 1.55, 1.56 and 1.57, one obtains:

$$v_r(r) = \frac{R}{r} \frac{dR}{dt} \tag{1.59}$$

We then obtain the following expression for the radial stress in the gel at the interface with the tissue:

$$\partial_t \sigma_{rr}(R) = -\frac{E_g}{1 + \nu_g} \times \frac{1}{R} \frac{\mathrm{d}R}{\mathrm{d}t}. \tag{1.60}$$

That leads to the following relation between the stresses just after and just before the steps of pressure:

$$\sigma_{rr}(R, t_c^+) = -\frac{E_g}{1+\nu_g} \ln \frac{R(t_c^+)}{R(t_c^-)}, \tag{1.61}$$

$$\sigma_{rr}(R, 0^+) = \sigma_{rr}(R, 0^-) - \frac{E_g}{1+\nu_g} \ln \frac{R(0^+)}{R(0^-)}, \tag{1.62}$$

where the stress in the gel is assumed to vanish before confluence.

Based on experimental data (Extended Data Fig. 5Ciii), one finds $\tau_g D_t \sim \tau_g (\mathrm{d}R/\mathrm{d}t)/R \approx 3.10^{-5} \ll 1$ for the dynamics between tissue confluence and the increase of pressure and $\tau_g D_t \sim \tau_g (\mathrm{d}R/\mathrm{d}t)/R \approx 6.10^{-4} \ll 1$ during the dynamics after the initial step of pressure. This indicates that the effect of the material derivative in Eq. 1.53 can be neglected and the gel behaves as a viscous fluid:

$$\sigma_{\alpha\beta} = \mu_g \left(v^g_{\alpha\beta} + \frac{\nu_g}{1-2\nu_g} v^g_{\gamma\gamma} \delta_{\alpha\beta} \right). \tag{1.63}$$

Using Eqs. 1.63, 1.55, 1.56 and 1.57, and integrating between R and ∞ (with $\sigma_{rr}(\infty) = 0$), ones obtains:

$$\sigma_{rr}(R) = -\mu_g \frac{1}{R} \frac{\mathrm{d}R}{\mathrm{d}t}. \tag{1.64}$$

Taking the highest values of τ_g ($64s$) and E_g ($1700Pa$) computed from stress relaxation experiments (Extended Data Fig. 1Bii, 5Ciii) and $\nu_g = 0.3$ ([15, 16]), we numerically solved the system of Eqs. 1.52, 1.23, 1.24 and 1.25 with the expressions of the stress at the boundary gel $\sigma_{rr}(R)$ discussed above (Eqs. 1.64, 1.61, 1.62). The effect of the gel on the dynamics of tube radius and actin mean orientation turns out to be small (Extended Data Fig. 5Ci,ii). Simulating the dynamics of this system with no actin elasticity ($K_a = 0$) results in an explosion of the radius, showing that the gel contribution can not explain slowing down over time of the tube expansion (Extended Data Fig. 5Ci,ii).

1.3.4 Elastic behaviour of residual tension

The constitutive equation on residual tension (Eq. 1.19) corresponds to a viscoelastic response. The short-time elastic response of t^r_{ij} ensures that just after pressure application, the tissue elongation in the circumferential direction leads to higher tension in that direction, in agreement with laser ablation experiments (Fig. 1Bii). We find that these elastic stresses t^r_{ij} also have to relax to explain experimental data: indeed the reorientation of actin fibers after pressure increase leads to higher circumferential tension and would lead to a decrease in the tube radius, if the residual elastic tension had not relaxed (Extended Data Fig. 5Di).

We also tested the possibility that the residual tension t^r_{ij} responds elastically, while the elastic stresses in actin fibers t^a_{ij} relax over time. To test this possibility, we fitted the experimental time evolution of actin elongation by an exponential function $q(t) = a + b(1 - e^{-ct})$ (Extended Data Fig. 5Dii), and numerically solved the evolution of the tube radius for a purely elastic response of the residual tension:

$$t^{r\,\theta}_{\ \theta} = K \ln(R/R_0), \tag{1.65}$$

with K an elastic modulus and R_0 the value of the radius before pressure increase. We also assumed that t^a_{ij} follows the viscoelastic constitutive equation discussed in section 1.3.2 (Eqs. 1.49 and 1.50) with $\sigma_a(0^-) = 0$. We fitted the radius experimental value by finding optimal values of the parameters through a Nelder-Mead algorithm to minimize the following cost function:

$$C(\{p\}) = \sum_{i=1}^{N_{bin}} \left(\frac{R(\{p\}, t_i) - R_{bin}(t_i)}{R_{bin}(t_i)} \right)^2, \tag{1.66}$$

with R_{bin} as defined in section 1.3.2 of this SI. The model we obtain then reproduced correctly the radius dynamics (Extended Data Fig. 5Di), but also predicted higher longitudinal tension just after pressure increase (Extended Data Fig. 5Diii), in contradiction with results from laser ablation experiments (Fig. 1Bii).

1.3.5 Effect of tube curvature on actin stress fibers orientation

We tested the effect of adding a curvature coupling term $-\lambda_c \tilde{C}_{ij}$ in Eq. 1.11. Such a term would correspond to actin fibers orienting along the axis of minimal curvature, possibly through bending energy minimization. This could explain why actin fibers preferentially align along the tube axis before the application of the higher pressure. Going through the fitting procedure with this additional term, we find a small dimensionless value $\hat{\lambda}_c = \tau\lambda_c/R_0 = 2.10^{-4} \ll 1$. This suggests that the effect of curvature on fiber alignment is small compared to other effects, but is sufficient to select a preferred orientation $q = -q_0$ at tissue confluence.

1.3.6 Coupling of actin stress fiber orientation dynamics on total tension

Here we discuss the coupling term with tension in the dynamics of actin mean orientation (Eq. 1.11). We assumed that this coupling depends on t^r_{ij}, the residual tension of the tissue without actin. We discuss here an alternative choice, introducing a coupling to the total tissue tension t_{ij}:

$$D_t q_{ij} = -\gamma \left(\frac{1}{2} q_{kl} q^{kl} - q_0^2\right) q_{ij} + \beta \tilde{t}_{ij}, \tag{1.67}$$

with $\tilde{t}_{ij} = t_{ij} - t^k_k g_{ij}/2$. The constitutive equations for t^a_{ij} and t^r_{ij}, Eqs. 1.16, 1.17 and 1.19, are unchanged. The dimensionless system of equations then becomes:

$$R\Delta P = \sigma + (1 + K_a s)\left(q + \frac{1}{2}\right), \tag{1.68}$$

$$\left(1 + \frac{\mathrm{d}}{\mathrm{d}t}\right)\sigma = \frac{\mu}{R}\frac{\mathrm{d}R}{\mathrm{d}t}, \tag{1.69}$$

$$\frac{\mathrm{d}s}{\mathrm{d}t} = \left(q + \frac{1}{2}\right)\frac{1}{R}\frac{\mathrm{d}R}{\mathrm{d}t}, \tag{1.70}$$

$$\frac{\mathrm{d}q}{\mathrm{d}t} = -\gamma\left(q^2 - q_0^2\right) q + \beta\left[\sigma/2 + (1 + K_a s) q\right]. \tag{1.71}$$

We then looked for the optimal set of parameters for this model to account for experimental data, solving the dynamics of the system as described in sections 1.1.6 and 1.1.7 and using a Nelder-Mead algorithm to minimize the cost function defined in Eq. 1.38. This version of the model still shows good agreement with experimental data (Extended Data Fig. 5Ei,ii). However, the radius evolution exhibits unexpected behaviour for pressures smaller than $\simeq$ 350Pa. Indeed for a small pressure increase at $t = 0$, the initial circumferential tension anisotropy is not sufficient to drive actin fibers orientation circumferentially before t^r_{ij} relaxes. The residual tension then decreases, the fibers are still longitudinal and, as the total tension arises mostly from actin stress fibers, the total tension anisotropy becomes longitudinal (Extended Data Fig. 5Eiii). As a result, q decreases (Extended Data Fig. 5Eii), meaning that fibers become more longitudinal. The actin network is thus not able to resist the circumferential tube expansion, and the tube radius significantly increases (Extended Data Fig. 5Ei). This leads to a new increase in t^r_{ij}, the tension anisotropy becomes circumferential, allowing at last fibers to reorient circumferentially and the radius to stabilize to a steady value that is higher than steady-state values reached for higher pressure (Extended Data Fig. 5Ei).

We also observe that at intermediate pressures between 150 Pa and 650Pa, the model with mechanosensing coupling on t^r_{ij} exhibits a non-monotonous tube radius expansion as a function of pressure, due to a delay in fiber reorientation. The effect is however less pronounced that for the coupling with t_{ij}. As we lack experimental data at these intermediate pressures, we can not conclude at this point between the two models.

1.3.7 Model of the tissue as an isotropic, viscoelastic material

In the model, the tension generated inside the tissue is the combination of an active and an elastic tension, oriented along actin stress fibers, and a residual viscoelastic response (Eqs 1.16, 1.17 and 1.19). Here we ask if a model where the tissue effectively behaves as an isotropic material, and in particular where tension is not oriented along actin stress fibers, can still explain experimental measurements. Therefore we consider the

tension in the tissue to be given by a Zener model (combination of a Maxwell model in parallel with a spring, Extended Data Fig. 5Fiv):

$$t_{ij} = t^r_{ij} , \tag{1.72}$$

$$t^r_{ij} = t^{el}_{ij} + t^{ve}_{ij} , \tag{1.73}$$

$$D_t t^{el}_{ij} = K v_{ij} , \tag{1.74}$$

$$(1 + \tau D_t) t^{ve}_{ij} = \mu v_{ij} . \tag{1.75}$$

We assume that for $R = R_c$ the tension t^{el}_{ij} vanishes, $t^{el}_{ij} = 0$. In this subsection, we use dimensional equations. This results in the following equation for the tube radius:

$$\left(1 + \tau \frac{\mathrm{d}}{\mathrm{d}t}\right)(R\Delta P) = \frac{\mu + \tau K}{R} \frac{\mathrm{d}R}{\mathrm{d}t} + K \ln\left(\frac{R}{R_c}\right) . \tag{1.76}$$

We looked for optimal parameters solving the dynamics of this system as described in sections 1.1.6 and 1.1.7 and using a Nelder-Mead algorithm to minimize the following cost function

$$C(\{p\}) = A \times \left[R(\{p\}, t_c^-) - R_c\right]^2 + \sum_{i=1}^{N_R} \left(\frac{R(\{p\}, t_i/\tau) - \bar{R}_i/R_0}{\bar{R}_i/R_0}\right)^2 . \tag{1.77}$$

The time evolution of the radius obtained from this fitted procedure exhibits a different qualitative behaviour than the mean curve of radius evolution experimental points (Extended Data Fig. 5Fi), showing an inverse concavity compared to experimental data.

Additional active isotropic tension. We now consider the same model, but adding an isotropic active tension $\zeta_0 g_{ij}$. The constitutive equations of the model become:

$$t_{ij} = t^r_{ij} + \zeta_0 g_{ij} , \tag{1.78}$$

$$t^r_{ij} = t^{el}_{ij} + t^{ve}_{ij} , \tag{1.79}$$

$$D_t t^{el}_{ij} = K v_{ij} , \tag{1.80}$$

$$(1 + \tau D_t) t^{ve}_{ij} = \mu v_{ij} , \tag{1.81}$$

which leads to the following equation for the tube radius evolution:

$$\left(1 + \tau \frac{\mathrm{d}}{\mathrm{d}t}\right)(R\Delta P - \zeta_0) = \frac{\mu + \tau K}{R} \frac{\mathrm{d}R}{\mathrm{d}t} + K \ln\left(\frac{R}{R_c}\right) , \tag{1.82}$$

We then look for optimal parameters solving the dynamics of this system as described in sections 1.1.6 and 1.1.7 and using a Nelder-Mead algorithm to minimize the cost function defined in Eq. 1.77. A better agreement is found with experimental data (Extended Data Fig. 5Fi), as expected from the addition of an extra parameter. However, the radius obtained from the model reaches a plateau about 24 hours after pressure increase, in contrast to experiments where the radius keeps increasing during that period.

1.3.8 Isotropic viscoelasticity with anisotropic active tension

Here we ask whether a model where the long-time scale elastic response is dependent on tissue deformation, but not on the orientation of actin stress fibers, can still account for experimental data. We consider the same Zener model than in the previous section, with the addition of an active anisotropic tension $\zeta_0 n_{ij}$ oriented along actin fibers. The constitutive equations then become (here using dimensional parameters):

$$t_{ij} = t^a_{ij} + t^r_{ij} , \tag{1.83}$$

$$t^a_{ij} = \zeta_0 n_{ij} , \tag{1.84}$$

$$t^r_{ij} = t^{el}_{ij} + t^{ve}_{ij} \ , \tag{1.85}$$

$$D_t t^{el}_{ij} = K v_{ij} \ , \tag{1.86}$$

$$(1 + \tau D_t) t^{ve}_{ij} = \mu v_{ij} \ , \tag{1.87}$$

$$D_t q_{ij} = -\gamma \left(\frac{1}{2} q_{kl} q^{kl} - q_0^2 \right) q_{ij} + \beta \tilde{t}^r_{ij} \ . \tag{1.88}$$

This results in the following coupled dynamics for the tube radius and actin nematic q:

$$\left(1 + \tau \frac{\mathrm{d}}{\mathrm{d}t}\right) \left(R\Delta P - \zeta_0 \left(q + \frac{1}{2} \right) \right) = \frac{\mu + \tau K}{R} \frac{\mathrm{d}R}{\mathrm{d}t} + K \ln \left(\frac{R}{R_c} \right), \tag{1.89}$$

$$\frac{\mathrm{d}q}{\mathrm{d}t} = -\gamma \left(q^2 - q_0^2 \right) q + \frac{1}{2} \beta \left(R\Delta P - \zeta_0 (q + \frac{1}{2}) \right), \tag{1.90}$$

We optimized the parameters of this model solving the dynamics of this system as described in sections 1.1.6 and 1.1.7 and using a Nelder-Mead algorithm to minimize the cost function introduced in Eq. 1.38. The model is in good agreement with experimental data for radius and actin stress fiber nematic for $t > 0$ (Extended Data Fig. 5Fi,ii). We conclude that assuming an active tension oriented along actin fibers allows to reproduce experimental data, with long time scale elasticity oriented or not along actin fibers.

<table>
<tr><th>Actin mechanosensing</th><th>Residual tension</th><th>Actin oriented tension</th><th>Results</th></tr>
<tr><td rowspan="9">Residual tension</td><td rowspan="2">Purely elastic</td><td>Purely elastic
+
Active tension</td><td>Radius decrease after initial jump
(Sec. 1.3.4, Extended Data Fig. 5Di,
green curve)</td></tr>
<tr><td rowspan="2">Viscoelastic
+
Active tension</td><td>Tension anisotropy after pressure
increase disagrees with experiment
(Sec. 1.3.4, Extended Data Fig. 5Di, red curve,
Extended Data Fig. 5Diii)</td></tr>
<tr><td rowspan="4">Viscoelastic</td><td>Good agreement
with experimental data
(Sec. 1.3.2, Extended Data Fig. 5A)</td></tr>
<tr><td>Purely elastic
+
Active tension</td><td>Good agreement
with experimental data
(Sec. 1.1.6, Fig. 4D)</td></tr>
<tr><td>Active tension
+
Viscoelastic gel</td><td>Fast explosion of the radius
(Sec. 1.3.3, Extended Data Fig. 5C,
dashed green curves)</td></tr>
<tr><td>Purely elastic
+
Active tension
+
Viscoelastic gel</td><td>Similar results to the main model
(Sec. 1.3.3, Extended Data Fig. 5C,
dashed green curves)</td></tr>
<tr><td>Zener model</td><td rowspan="2">None</td><td>Dynamics of radius does not
agree with experimental data
(Sec. 1.3.7, Extended Data Fig. 5Fi,
green curve)</td></tr>
<tr><td>Zener model
+
Isotropic active
tension</td><td>Radius plateau at 24hours
(Sec. 1.3.7, Extended Data Fig. 5Fi,
dashed red curve)</td></tr>
<tr><td>Zener model</td><td>Active tension</td><td>Good agreement for radius and actin
orientation dynamics
(Sec. 1.3.8, Extended Data Fig. 5F)</td></tr>
<tr><td>Total tension</td><td>Viscoelastic</td><td>Purely elastic
+
Active tension</td><td>Dramatic radius increase at low
pressures (Sec. 1.3.6, Extended Data Fig. 5E)</td></tr>
</table>

Table 1.4: Summary of model variations. Choices corresponding to the main model are indicated in bold.

Part 2 Mechanical model of the endothelial tube instantaneous strain-stiffening

In the following, we present the isotropic nonlinear elastic Gent model, that we then use to characterize the deformation of the endothelial tubes under applied pressure. The contribution of the deformed collagen material surrounding the tube is accounted for, to determine the pressure differential across the cell layer. The experimental data are fit to this model with an orthogonal distance regression, to determine elastic moduli and maximum extension of the nonlinear tissue. The effect of the collagen concentration on the tissue properties are investigated, and the contribution of the actin cytoskeleton and cell-cell junctions are discussed.

2.1 Model of the endothelial tube

2.1.1 Gent model

The cell monolayer is modeled as a nonlinear elastic cylindrical shell, using the Gent model, surrounded by a linear elastic collagen medium [17, 18]. The Gent model is an empirical model for hyperelasticity, that captures the quasi-linear response at small strains and strain hardening behavior at larger strain. The model relates the pressure drop across the cell layer as a function of the extension of the vessel radius, defined as $\lambda = \frac{R}{R_0}$:

$$\Delta P = -\frac{hJ_m\left(\lambda^4 - 1\right)G}{R_0\lambda^2(1-(2+J_m)\lambda^2+\lambda^4)} \tag{2.1}$$

where h is the monolayer thickness, G the shear elastic modulus and J_m the extensibility parameter. Assuming the material is isotropic, the shear modulus can then be used to compute the Young's modulus by

$$E = 2G(1+\nu), \tag{2.2}$$

where ν is the Poisson's ratio of the endothelial tissue, assumed to be 0.5; while the extensibility can be used to define a maximum extension by

$$\lambda_{max} = \sqrt{(2+J_m+\sqrt{J_m}\sqrt{4+J_m})/2}. \tag{2.3}$$

2.1.2 Contribution of the surrounding collagen gel

In our experiments, both the cell monolayer and the surrounding collagen resist deformation induced by the applied luminal pressure, P. The total pressure drop between the lumen and the outside applied pressure equals the sum of the pressure drop across the cell layer, ΔP, and across the collagen matrix, P_{gel}. We are interested in the elastic response of the cell layer, which depends on the pressure drop across the layer. The pressure drop across the cell layer is then $\Delta P = P - P_{gel}$.

To determine the pressure at the interface between the cell layer and collagen, we need to account for the deformation of the collagen. We model the collagen as a linear elastic material surrounding a cylindrical inclusion. The circumferential strain of the cylinder results in a pressure at surface of the surrounding collagen, that decays to a zero reference pressure far from the cylinder. The pressure exerted by the surrounding collagen is determined using the following equation:

$$\Delta P_c = \frac{E_g\epsilon}{(1+\nu_g)} \tag{2.4}$$

where E_g is the Young's modulus of the collagen and the strain is $\epsilon = \lambda - 1$. The Poisson's ratio of the collagen gel ν_g is assumed to be 0.3 [15, 16].

The elastic modulus of collagen varies significantly with collagen concentration, following a power law [19]. The modulus of the 2 mg/mL collagen has been measured to be around 50 Pa and we measured the modulus of the 6 mg/mL collagen around 1 kPa (Extended Data Fig. 1Bii). The contribution of collagen to the pressure drop is calculated from Eq. 2.4 using the measured strain, allowing us to determine the pressure drop across the cell layer. The contribution of the collagen gel to the pressure drop is largest at small deformation, but is still small compared to the cell layer [18]. Assuming a linear elastic response of the cell layer and taking approximately $P = 900$Pa, $\epsilon = 10\%$ (Fig. 1Ci) and $E_g = 1$kPa for the elastic modulus of the collagen gel at 10% strain after 100s of strain application (Extended Data Fig. 1Bii), the relative contribution of the collagen layer to the pressure drop is approximately $P_{gel}/P < 10\%$ for the 6 mg/mL collagen, and smaller for the 2 mg/mL collagen.

2.1.3 Fitting procedure

The thickness of the monolayer h is observed on fluorescent images of actin immunostainings to vary within the tissue, being maximal above cell nuclei and minimal in between nuclei. By delineating manually the monolayer contour on several images, the average thickness is measured to be $h = 3.6 \pm 0.5 \mu m$.

Using the continuous recording of the circumferential strain as a function of pressure (see Methods), the shear modulus G, extensibility parameter J_m, and reference state R_0, are determined by fitting individual strain-stress curve with orthogonal distance regression (ODR) using the Scipy package in Python [20]. The applied pressure is corrected with the gel pressure as discussed above. The fitted curves are shown in Fig. 1Ci, Ei, and the corresponding parameters are shown in Tables in the following section. The uncertainty is reported with the standard error on the fitted parameters.

2.2 Results

2.2.1 Substrate-dependent properties of endothelial tubes

We first sought to measure the mechanical properties of our endothelial tubes, and to check for potential substrate dependencies due to cellular mechanosensing. To that end, we templated cellular monolayers inside two different collagen concentrations, 2 mg/mL and 6 mg/mL, thereby tuning the substrate ridigity.

Reference state R_0 The experimental data from monolayers on both collagen concentrations displays a significant portion of quasi-linear deformation, whose fit can be extrapolated to infer the reference state R_0 corresponding to the radius at $\Delta P = 0$. This value is found to be slightly above the needle radius (60μm) used to fabricate the channels, $R_0 = 62.5\mu$m, matching the diameter of $125 \mu m$ of bare channels reported in our previous work [21].

Young's modulus E The elastic moduli characterize the linear mechanical response of the cell layer at low applied pressures. We find that the inferred elastic modulus changes significantly with surrounding collagen concentration, a signature of cellular mechanosensitivity. The measured modulus of the cell layer templated in 6 mg/mL collagen is 122 ± 24 kPa, significantly greater than that of the layer templated in 2 mg/mL collagen 28 ± 3 kPa. This difference does not come from the different collagen stiffnesses, as the contribution of the gel to the response is accounted for in the calculation. We hypothesize that the difference is due to reinforcement of the actin network into thick bundles of stress fibers anchored at large focal adhesions, triggered by mechanosensing of the collagen properties (see Main text, Fig. 1C-D).

Maximal extension λ_{max} The strain stiffening response is determined by the extensibility parameter J_m of the Gent model in Eq. 2.1, but is most easily understood by the asymptotic extension value of the maximum extension, λ_{max}. We find that the inferred maximum extension are 1.36 ± 0.06 and 1.23 ± 0.1 for the tissues on

Device #	Radius R_0 (μm)	Shear modulus G (kPa)	Young's modulus E_g (kPa)	Extensibility Parameter J_m	Maximum extension λ_{max}
1	63.2 ± 0.3	10.6 ± 0.5	31.8 ± 1.6	0.274 ± 0.008	1.295 ± 0.005
2	64.2 ± 0.3	9.3 ± 0.4	27.9 ± 1.0	0.413 ± 0.008	1.371 ± 0.004
3	62.4 ± 0.3	8.7 ± 0.4	26.3 ± 1.1	0.533 ± 0.013	1.429 ± 0.006

Table 2.1: Fitted parameters for endothelial tubes templated in 2 mg/mL collagen. Uncertainties represent standard error on the parameter estimates from ODR.

Device #	Radius R_0 (μm)	Shear modulus G (kPa)	Young's modulus E (kPa)	Extensibility Parameter J_m	Maximum extension λ_{max}
1	62.6 ± 0.2	49.4 ± 3.4	148.3 ± 10.1	0.151 ± 0.025	1.213 ± 0.019
2	60.4 ± 0.2	40.3 ± 3.4	121.0 ± 10.1	0.063 ± 0.006	1.133 ± 0.006
3	62.5 ± 0.2	32.9 ± 0.8	98.7 ± 10.4	0.391 ± 0.052	1.361 ± 0.027

Table 2.2: Fitted parameters for endothelial tubes templated in 6 mg/mL collagen. Uncertainties represent standard error on the parameter estimates from ODR.

the 2 mg/mL and 6 mg/mL collagen respectively. The two maximum extensions are not significantly different and could be defined by subcellular components which show no or a weaker adaptation to substrate properties.

2.2.2 Subcellular determinants of endothelium properties.

To probe the role of the actin cytoskeleton and cell-cell junctions in the tissue properties, we treated the monolayers on 2 mg/mL collagen with cytochalasinD, to depolymerize actin, and EDTA, to perturb adherens junctions, prior to the stress-strain curve measurement. Because both treatments significantly softened the tissue (see below), the circumferential strain was increased and the initial low pressure of 150 Pa was sufficient to exceed the linear regime (Fig. 1E). Therefore, the reference state $R_0 = 62.5\mu m$ determined above was used as a fixed parameter in the fits.

Actin depolymerization We find the Young's modulus of cytochalasinD-treated tissues to be 11 ± 2 kPa, significantly smaller than that of the untreated tissues, 28 ± 3 kPa. This confirms that the actin network is a major contributor to tissue elasticity. Interestingly, the maximum extension is inferred to be 1.33 ± 0.02, similar to the untreated tissues at 1.36 ± 0.06. This suggests that the actin network contribution to the maximum extension is minimal, in line with the observation that the maximum extension is not substrate-sensitive (see above). Other subcellular components are thus driving the strain-stiffening properties of tissues found at larger pressures, with intermediate filaments being a likely candidate [22, 23].

Device #	Shear modulus G (kPa)	Young's modulus E (kPa)	Extensibility Parameter J_m	Maximum extension λ_{max}
1	3.1 ± 0.1	9.4 ± 0.3	0.374 ± 0.005	1.351 ± 0.003
2	3.8 ± 0.1	11.4 ± 0.4	0.386 ± 0.006	1.358 ± 0.003
3	4.2 ± 0.2	12.6 ± 0.5	0.313 ± 0.006	1.318 ± 0.003

Table 2.3: Fitted parameters for for monolayers on 2 mg/mL collagen and treated with cytochalasinD. An initial radius of $R_0 = 62.5$ μm is assumed for all data. Uncertainties represent standard error on the parameter estimates from ODR.

Junctions perturbation We find the Young's modulus of EDTA-treated tissues to be 12±4 kPa, significantly smaller than that of the untreated tissues, 28 ± 3 kPa. This could be due to softer cells or to the stretch of the soft collagen between the now-disjointed cells. The maximum extension is inferred to be 1.53 ± 0.06, significantly larger than that of the control tissues, at 1.36 ± 0.06. Similarly, this could be due to a modification of the cell

Device #	Shear modulus G (kPa)	Young's modulus E (kPa)	Extensibility Parameter J_m	Maximum extension λ_{max}
1	3.1 ± 0.1	9.2 ± 0.2	0.906 ± 0.011	1.583 ± 0.004
2	4.9 ± 0.1	14.8± 0.4	0.672 ± 0.013	1.491 ± 0.005

Table 2.4: Fitted parameters for monolayers on 2 mg/mL collagen and treated with EDTA. An initial radius of $R_0 = 62.5\,\mu$m is assumed for all data.

properties or to the stretch of the collagen gel.

Bibliography

[1] R. Etournay, M. Popović, M. Merkel, A. Nandi, C. Blasse, B. Aigouy, H. Brandl, G. Myers, G. Salbreux, F. Jülicher, et al., "Interplay of cell dynamics and epithelial tension during morphogenesis of the drosophila pupal wing," Elife, vol. 4, p. e07090, 2015.

[2] M. Popović, A. Nandi, M. Merkel, R. Etournay, S. Eaton, F. Jülicher, and G. Salbreux, "Active dynamics of tissue shear flow," New Journal of Physics, vol. 19, no. 3, p. 033006, 2017.

[3] A. C. Davison and D. V. Hinkley, Bootstrap Methods and their Application. Cambridge Series in Statistical and Probabilistic Mathematics, Cambridge University Press, 1997.

[4] J. Duque, A. Bonfanti, J. Fouchard, L. Baldauf, S. R. Azenha, E. Ferber, A. Harris, E. Barriga, A. Kabla, and G. Charras, "Rupture strength of living cell monolayers," bioRxiv, 2023.

[5] A. Marin-Llaurado, S. Kale, A. Ouzeri, T. Golde, R. Sunyer, A. Torres-Sanchez, E. Latorre, M. Gomez-Gonzalez, P. Roca-Cusachs, M. Arroyo, and X. Trepat, "Mapping mechanical stress in curved epithelia of designed size and shape," Nature Communications 2023 14:1, vol. 14, pp. 1–11, 7 2023.

[6] A. R. Harris, L. Peter, J. Bellis, B. Baum, A. J. Kabla, and G. T. Charras, "Characterizing the mechanics of cultured cell monolayers," Proceedings of the National Academy of Sciences, vol. 109, no. 41, p. 16449â16454, 2012.

[7] J. Pourati, A. Maniotis, D. Spiegel, J. L. Schaffer, J. P. Butler, J. J. Fredberg, D. E. Ingber, D. Stamenovic, and N. Wang, "Is cytoskeletal tension a major determinant of cell deformability in adherent endothelial cells?," American Journal of Physiology - Cell Physiology, vol. 274, 1998.

[8] T. Takemasa, K. Sugimoto, and K. Yamashita, "Amplitude-dependent stress fiber reorientation in early response to cyclic strain," Experimental Cell Research, vol. 230, pp. 407–410, 2 1997.

[9] T. Iba and B. E. Sumpio, "Morphological response of human endothelial cells subjected to cyclic strain in vitro," Microvascular Research, vol. 42, pp. 245–254, 11 1991.

[10] K. Hayakawa, N. Sato, and T. Obinata, "Dynamic reorientation of cultured cells and stress fibers under mechanical stress from periodic stretching," Experimental Cell Research, vol. 268, pp. 104–114, 8 2001.

[11] R. Krishnan, E. P. Canović, A. L. Iordan, K. Rajendran, G. Manomohan, A. P. Pirentis, M. L. Smith, J. P. Butler, J. J. Fredberg, and D. Stamenović, "Fluidization, resolidification, and reorientation of the endothelial cell in response to slow tidal stretches," American Journal of Physiology-Cell Physiology, vol. 303, no. 4, pp. C368–C375, 2012. PMID: 22700796.

[12] R. Kaunas, P. Nguyen, S. Usami, and S. Chien, "Cooperative effects of rho and mechanical stretch on stress fiber organization," Proceedings of the National Academy of Sciences of the United States of America, vol. 102, pp. 15895–15900, 11 2005.

[13] M. Gong, H. Yang, S. Zhang, Y. Yang, D. Zhang, Y. Qi, and L. Zou, "Superparamagnetic core/shell goldmag nanoparticles: Size-, concentration- and time-dependent cellular nanotoxicity on human umbilical vein endothelial cells and the suitable conditions for magnetic resonance imaging," Journal of Nanobiotechnology, vol. 13, 3 2015.

[14] J. Vasiceek, A. Balazi, M. Bauer, A. Svoradova, M. Tirpakova, M. Tomka, and P. Chrenek, "Molecular profiling and gene banking of rabbit epcs derived from two biological sources," Genes, vol. 12, pp. 1–27, 3 2021.

[15] D. M. Knapp, V. H. Barocas, A. G. Moon, K. Yoo, L. R. Petzold, and R. T. Tranquillo, "Rheology of reconstituted type I collagen gel in confined compression," Journal of Rheology, vol. 41, pp. 971–993, 09 1997.

[16] B. A. Lane, K. A. Harmon, R. L. Goodwin, M. J. Yost, T. Shazly, and J. F. Eberth, "Constitutive modeling of compressible type-i collagen hydrogels," Medical Engineering & Physics, vol. 53, pp. 39–48, 2018.

[17] A. Gent, "A new constitutive relation for rubber," Rubber chemistry and technology, vol. 69, no. 1, pp. 59–61, 1996.

[18] P. F. Salipante, S. D. Hudson, and S. Alimperti, "Blood vessel-on-a-chip examines the biomechanics of microvasculature," Soft Matter, vol. 18, no. 1, p. 117â125, 2022.

[19] K. A. Jansen, A. J. Licup, A. Sharma, R. Rens, F. C. MacKintosh, and G. H. Koenderink, "The role of network architecture in collagen mechanics," Biophysical Journal, vol. 114, p. 2665, 6 2018.

[20] P. T. Boggs and J. E. Rogers, "Orthogonal distance regression," Contemporary mathematics, vol. 112, pp. 183–194, 1990.

[21] C. A. Dessalles, C. Ramon-Lozano, A. Babataheri, and A. I. Barakat, "Luminal flow actuation generates coupled shear and strain in a microvessel-on-chip," Biofabrication, vol. 14, no. 1, p. 015003, 2021.

[22] E. Latorre, S. Kale, L. Casares, M. Gomez-Gonzalez, M. Uroz, L. Valon, R. V. Nair, E. Garreta, N. Montserrat, A. del Campo, and et al., "Active superelasticity in three-dimensional epithelia of controlled shape," Nature, vol. 563, no. 7730, p. 203â208, 2018.

[23] A. Singh, T. Saha, I. Begemann, A. Ricker, H. NÃ¼sse, O. Thorn-Seshold, J. Klingauf, M. Galic, and M. Matis, "Polarized microtubule dynamics directs cell mechanics and coordinates forces during epithelial morphogenesis," Nature Cell Biology, vol. 20, no. 10, p. 1126â1133, 2018.
