# Supplementary figures and images for: Interplay of actin nematodynamics and anisotropic tension controls endothelial mechanics
Source: Nat Phys. 2025 Apr 18;21(6):999–1008. doi: 10.1038/s41567-025-02847-3 (PMC12176649; doi:10.1038/s41567-025-02847-3)

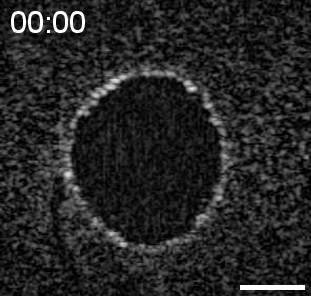

Supplement: Supplementary file 3 — Time-lapse images of the channel cross-section under a rapid pressure increase from 150 Pa to 650 Pa, showing an elastic expansion followed by a stable radius. Time, seconds. Scale bar, 50 µm. [file 41567_2025_2847_MOESM3_ESM.gif]

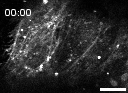

Supplement: Supplementary file 4 — Laser ablation experiment: fluorescence time-lapse images of LifeAct-endothelial cells on a soft collagen gel showing the endothelial actin network before and after longitudinal ablation, with a rapid opening of the wound, characteristic of high tissue tension in the circumferential direction. Time, seconds. Scale bar, 20 µm. [file 41567_2025_2847_MOESM4_ESM.gif]

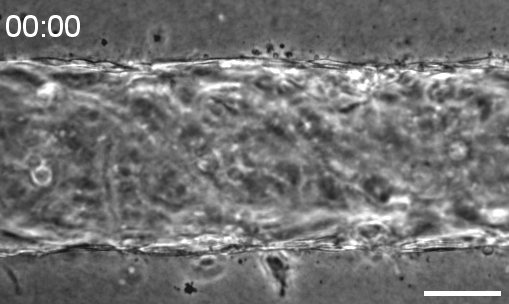

Supplement: Supplementary file 5 — Monolayer stiffness measurement: bright-field time-lapse images of an endothelial tube on a soft gel subjected to a linear increase in pressure from 150 Pa to 1,000 Pa, undergoing circumferential expansion with a visible slow-down characteristic of a strain-stiffening behaviour. Time, seconds. Scale bar, 50 µm. [file 41567_2025_2847_MOESM5_ESM.gif]

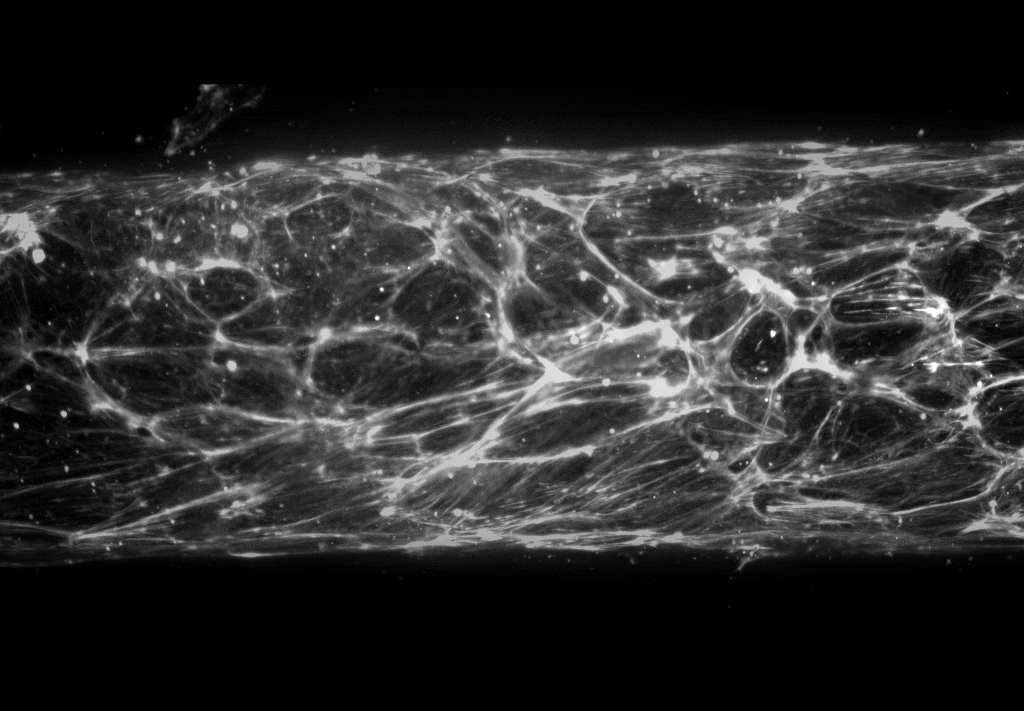

Supplement: Supplementary file 6 — Three-dimensional reconstruction of the actin network of an endothelial tube at 150 Pa. Obtained from a z stack of fluorescence images of a monolayer stained with phalloidin, showing a longitudinal alignment of the actin stress fibres. [file 41567_2025_2847_MOESM6_ESM.gif]

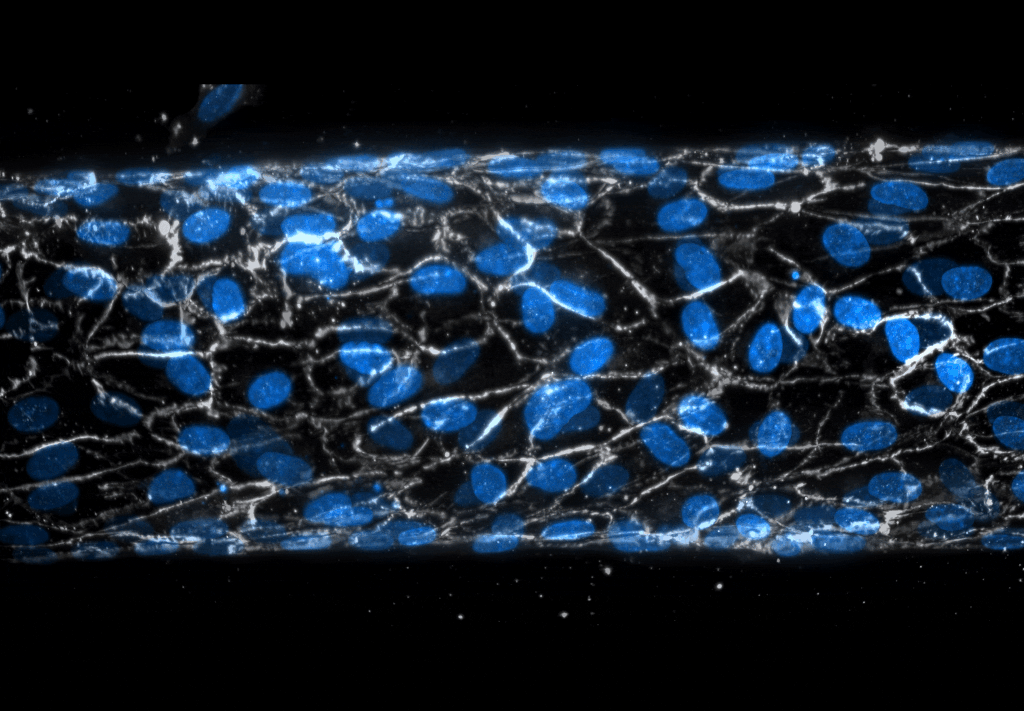

Supplement: Supplementary file 7 — Three-dimensional reconstruction of the cells and nuclei of an endothelial tube at 150 Pa. Obtained from a z stack of fluorescence images of a monolayer stained with VE-cadherin (white) and DAPI (cyan), showing a longitudinal alignment of the cells and nuclei. [file 41567_2025_2847_MOESM7_ESM.gif]

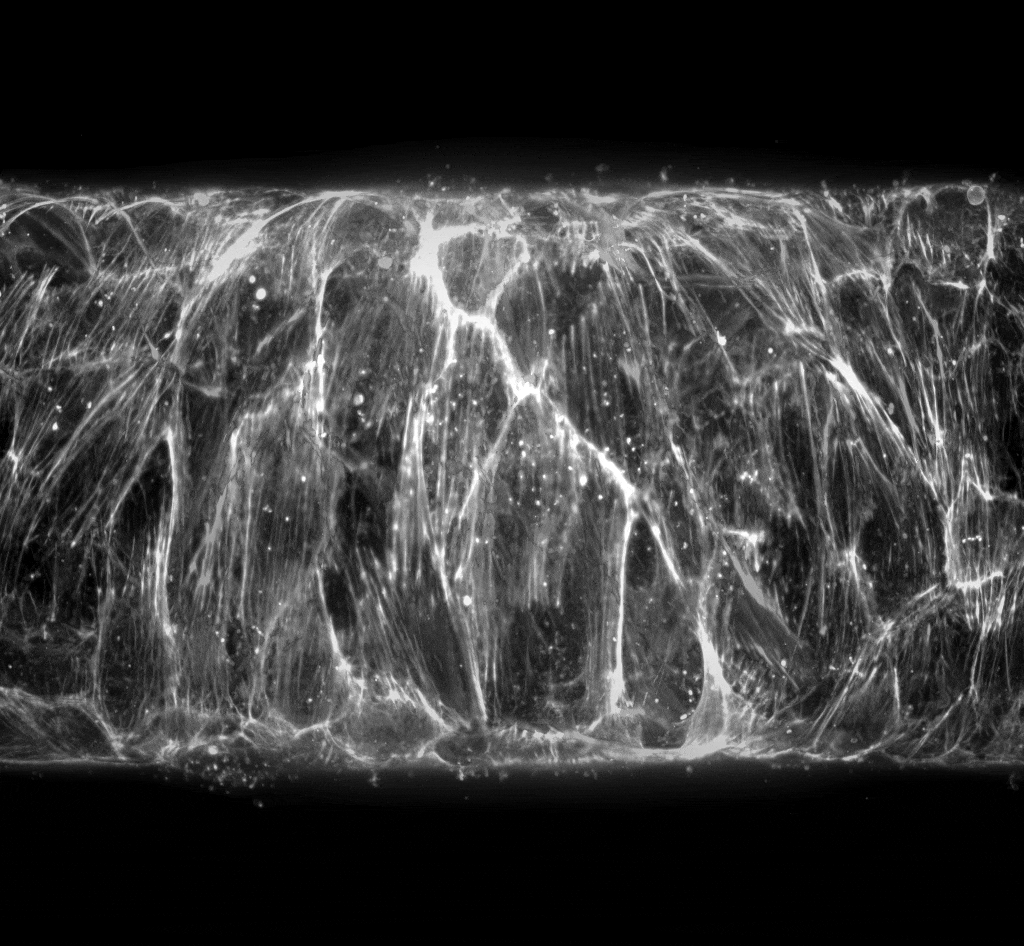

Supplement: Supplementary file 8 — Three-dimensional reconstruction of the actin network of an endothelial tube at 650 Pa. Obtained from a z stack of fluorescence images of a monolayer stained with phalloidin, 7 h after pressure increase, showing a circumferential alignment of the actin stress fibres. [file 41567_2025_2847_MOESM8_ESM.gif]

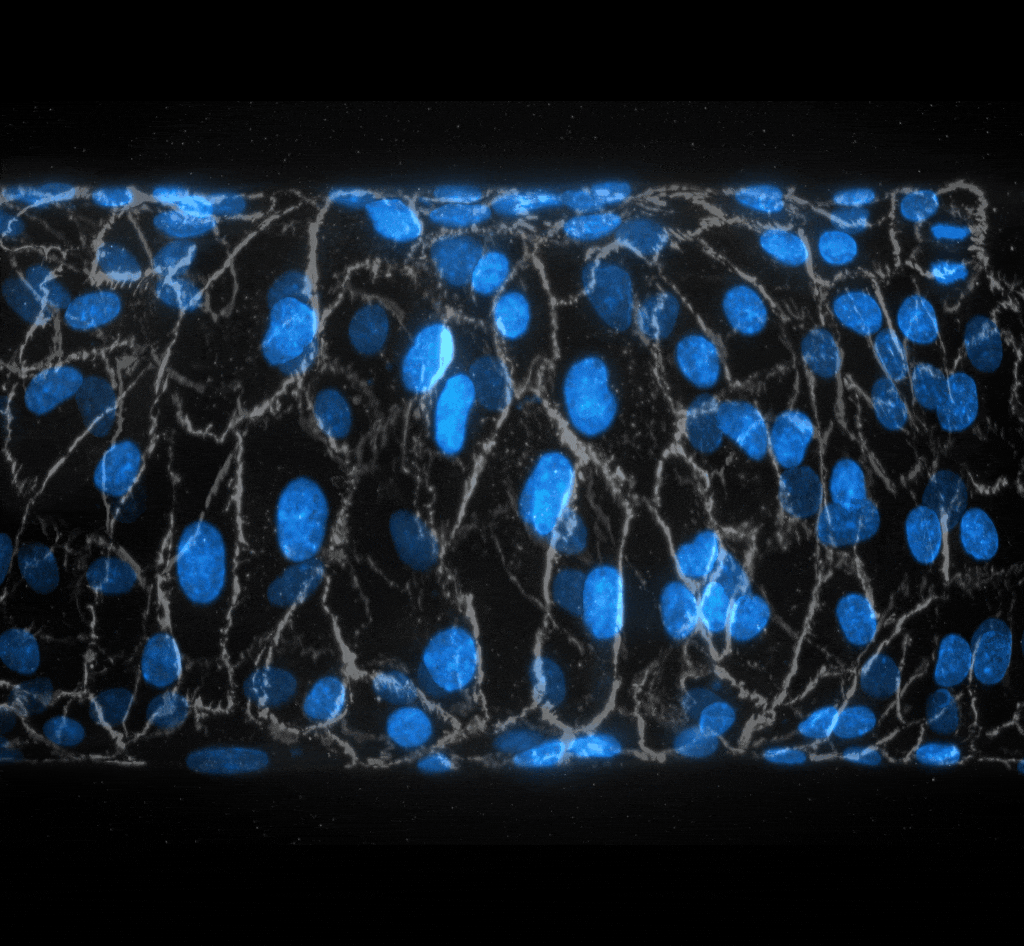

Supplement: Supplementary file 9 — Three-dimensional reconstruction of the cells and nuclei of an endothelial tube at 650 Pa. Obtained from a z stack of fluorescence images of a monolayer stained with phalloidin, 7 h after pressure increase, showing a circumferential alignment of the cells and nuclei. [file 41567_2025_2847_MOESM9_ESM.gif]

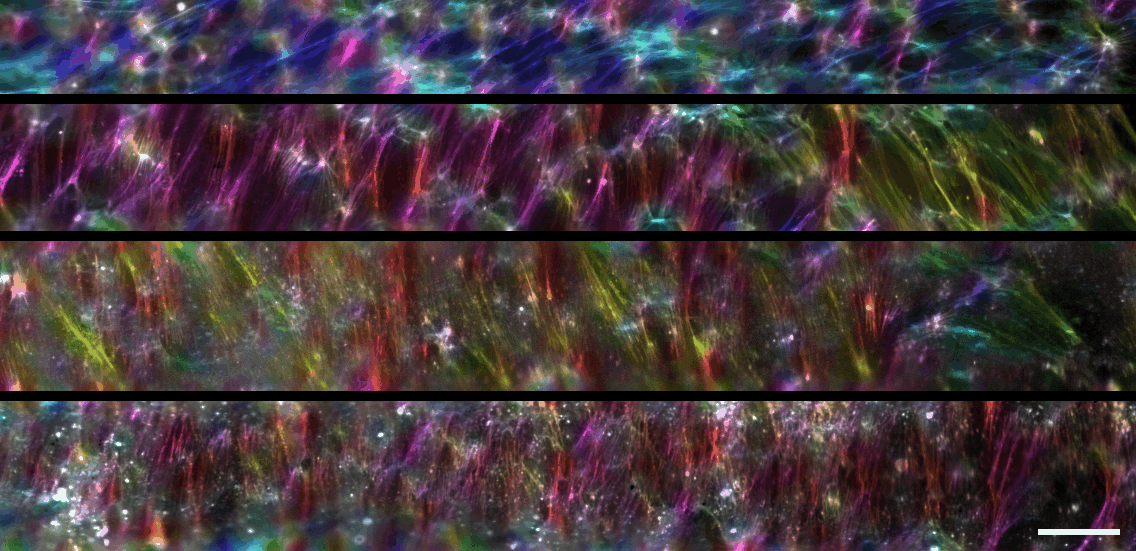

Supplement: Supplementary file 10 — Variations in the actin nematic field along the channel length. Endothelial tubes stained with phalloidin, with the orientation of the actin stress fibres colour coded, at 0 h (top row), 7 h (second row), 24 h (third row) and 56 h (bottom row), showing the variability in fibre distribution along the channel length (each frame in the video is taken at a different x position). [file 41567_2025_2847_MOESM10_ESM.gif]
